# Comparative analysis of the chimpanzee and human brain superficial structural connectivities

**DOI:** 10.1007/s00429-024-02823-2

**Published:** 2024-07-17

**Authors:** Maëlig Chauvel, Marco Pascucci, Ivy Uszynski, Bastien Herlin, Jean-François Mangin, William D. Hopkins, Cyril Poupon

**Affiliations:** 1 BAOBAB, NeuroSpin, Paris-Saclay University, CNRS, CEA, Gif-sur-Yvette, France; 2 Department of Neurophysics, Max Planck Institute for Human Cognitive and Brain Sciences, Leipzig, Germany; 3 Rehabilitation Unit, AP-HP, Pitié-Salpêtrière Hospital, Paris, France; 4 Department of Comparative Medicine, Michale E Keeling Center for Comparative Medicine and Research, The University of Texas MD Anderson Cancer Center, Bastrop, TX USA

**Keywords:** Short association fibers, Isomap, Clustering, Chimpanzee connectivity, Diffusion MRI

## Abstract

Diffusion MRI tractography (dMRI) has fundamentally transformed our ability to investigate white matter pathways in the human brain. While long-range connections have extensively been studied, superficial white matter bundles (SWMBs) have remained a relatively underexplored aspect of brain connectivity. This study undertakes a comprehensive examination of SWMB connectivity in both the human and chimpanzee brains, employing a novel combination of empirical and geometric methodologies to classify SWMB morphology in an objective manner. Leveraging two anatomical atlases, the Ginkgo Chauvel chimpanzee atlas and the Ginkgo Chauvel human atlas, comprising respectively 844 and 1375 superficial bundles, this research focuses on sparse representations of the morphology of SWMBs to explore the little-understood superficial connectivity of the chimpanzee brain and facilitate a deeper understanding of the variability in shape of these bundles. While similar, already well-known in human U-shape fibers were observed in both species, other shapes with more complex geometry such as *6* and *J* shapes were encountered. The localisation of the different bundle morphologies, putatively reflecting the brain gyrification process, was different between humans and chimpanzees using an isomap-based shape analysis approach. Ultimately, the analysis aims to uncover both commonalities and disparities in SWMBs between chimpanzees and humans, shedding light on the evolution and organization of these crucial neural structures.

## Introduction

Diffusion MRI tractography (dMRI) represents the pioneering technique for investigating white matter pathways, encompassing not only the long, deep white matter bundles that have been extensively studied through methods such as Klinger’s dissections (Klingler and Ludwig 1956) but also the short-range white matter bundles, the exploration of which continues to be a focal point within the scientific community. In vivo studies of the human brain have predominantly focused on long-range connections, with relatively limited exploration of short-range connections so far. The delineation between deep white matter bundles (DWMB) and short white matter bundles (SWMBs) remains a topic of ongoing debate without a unified consensus within the scientific community. The comprehensive mapping of both anatomical and functional knowledge pertaining to SWMB is gaining increasing attention, driven by recent advancements in dMRI methodologies.

The exploration of SWMB connectivity in the human brain has been carried out through a combination of dMRI and brain dissection techniques (Guevara et al. 2020; Catani et al. 2012), and was assumed to correspond to sub-cortical white matter fibers connecting one gyrus to another (Meynert 1885; Oishi et al. 2008; Zhang et al. 2018; Shah et al. 2019). Although the precise functional role of these loco-regional connections remains uncertain, they are considered pivotal to the brain’s structural organization (Sporns and Honey 2006), and the efficiency of its functional networks (Meynert 1885). Their role has already been investigated with respect to age and gender (Phillips et al. 2013; Wu et al. 2014), brain lateralization (Catani et al. 2012; Magro et al. 2012), neurological disorders (Ji et al. 2019) such as schizophrenia (Nazeri et al. 2013; Phillips et al. 2011), Alzheimer’s disease (Fornari et al. 2012), Huntington’s disease (Phillips et al. 2016), autism (Sundaram et al. 2008; d’Albis et al. 2018) and age-correlated cognitive decline (Nazeri et al. 2015). Additionally, some studies have investigated particular brain regions, such as the precentral and postcentral regions (Gahm and Shi 2019; Magro et al. 2012), frontal areas (Catani et al. 2012; Conturo et al. 1999; Oishi et al. 2008), the occipital (Sachs 1892), and parietal lobes (Catani et al. 2017).

In addition to short U-fibers following the convexity of gyral convolutions (Meynert 1885), SWMBs encompass a broader spectrum of connections, spanning from U-fibers to extended loco-regional short association bundles that traverse multiple sulci, thereby exhibiting diverse morphologies and shapes. Therefore, we will not use the term "U-fibers" but prefer the generic term SWMBs covering a wider range of subcortical connections including U-fibers.

Some SWMB atlases of the human brain have already been released (Guevara Alvez 2011; Labra Avila 2020; Guevara et al. 2017), but fewer for the chimpanzee brain for which only two atlases have recently been published addressing both deep (Bryant et al. 2020) and superficial (Chauvel et al. 2023) white matter connectivity.

Given the significant inter-species differences in cortical folding patterns, comparing the networks of short fiber bundles that circumvent these folds presents a substantial challenge. Certain brain regions exhibit great variability when comparing humans and non-human primates, for instance at the level of the frontal lobe (Yeterian et al. 2012), making such comparisons challenging.

Previous pioneering studies have focused on comparing SWMBs between humans and monkeys, as seen in Oishi et al. (2011), who explored differences with the macaque brain, and in the study by Catani et al. (2017), investigating differences in SWMBs in the parietal lobe among humans, macaques, and vervet monkeys. However, all these studies investigated a limited number of subjects and did not perform quantitative analysis on the reported tracts, thus restricting the interpretability of intra-subject and inter-species variability and representativeness.

In this study, we propose a novel classification approach of the SWMBs morphology of the chimpanzee and human brains to provide a robust methodology to compare chimpanzee and human brain connectivity. To achieve this, we employed a first empirical approach, supplemented by a rigorous geometric methodology, to mitigate any potential bias stemming from subjective preconceptions. This latter approach builds upon the framework proposed by Sun et al. in 2017 (Sun et al. 2017), originally designed to analyze the shape of cortical sulci, adapted in the frame of this work.

The present analytical framework was employed for the comparative analysis of two anatomical atlases, namely the Ginkgo Chauvel chimpanzee atlas comprising 844 SWMBs and the Ginkgo Chauvel human atlas (Chauvel et al. 2023) comprising 1375 SWMBs. This analytical framework relies on utilizing the centroid of each SWMB as a sparsely encoded representation of the morphological characteristics of these bundles. This exploratory framework allowed us to explore in finer detail the still poorly understood superficial connectivity of the chimpanzee brain, in order to better understand the variability of the various observed bundle shapes. Ultimately, this novel framework was exploited to uncover both similarities and differences between chimpanzee and human brain superficial connectivities.

## Materials and methods

The chimpanzee atlas used for this study is the Ginkgo Chauvel chimpanzee atlas (Chauvel et al. 2022) for which a complete description can be found in Chauvel et al. (2023). The human atlas used and entirely designed for this study is the Ginkgo Chauvel human atlas (Chauvel et al. 2022) for which a complete description can be found in Chauvel (2023). Both atlases were established from comparable chimpanzee and human cohorts and using the same methodology to minimize any bias stemming from differences in methods.

A brief description is provided hereafter to summarize the eventual analyses.

### Chimpanzee and human cohorts

#### Chimpanzee cohort

We considered data coming from 39 healthy in vivo chimpanzees including 23 females and 16 males, imaged between 9 and 35 years old (mean = 19 years old) and housed at the Yerkes National Primate Research Center (YNPRC, Atlanta). Chimpanzee MRI scans were obtained from a data archive of scans acquired prior to the 2015 implementation of US Fish and Wildlife Service and National Institutes of Health regulations governing research with chimpanzees. All the scans reported in this publication were completed by the end of 2012 and have been used in previous studies (e.g. Vickery et al. 2020; Bryant et al. 2020; Chauvel et al. 2023).

Each individual was scanned on a 3 Tesla Trio MRI system (Siemens, Erlangen) using a birdcage coil with a dedicated imaging protocol comprising anatomical (0.625 mm isotropic spatial resolution) and diffusion data (1.9 mm isotropic spatial resolution, b = 1000 s/mm$^{2}$ single-shell acquisition with 60 diffusion directions, TE/TR = 86 ms/6 s, flip angle FA = 90$^{\circ }$, read bandwidth RBW = 1563 Hz/pixel, matrix size 128 x 128, FOV = 243.2 x 243.2 mm^2^.). All procedures were carried out in accordance with protocols approved by YNPRC and the Emory University Institutional Animal Care and Use Committee.

#### Human cohort

We used a cohort of 39 healthy human subjects stemming from the Human Connectome Project (HCP, release: http://www.humanconnectomeproject.org/) including 23 females and 16 males, imaged between 22 and 35 years old. The human cohort was intentionally built to match the chimpanzee cohort, with the same number of subjects and male/female ratio. It included for each subject a series of anatomical and diffusion-weighted MRI (dMRI) sequences performed on a Connectome Skyra 3T MRI system. The multiple-shell dMRI sequence was acquired using a 2D spin-echo single-shot multiband EPI sequence (multi-band factor of 3, monopolar diffusion gradient pulses, 1.25 mm isotropic spatial resolution, TR/TE = 5500/89.50 ms) over 3 shells at b = 1000/2000/3000 s/mm$^{2}$ along 90 diffusion directions for each shell, and 6 non-diffusion-weighted (b = 0 s/mm$^{2}$) reference images.

### Preprocessing and cortical parcellation

#### Chimpanzees

**Fig. 1:**
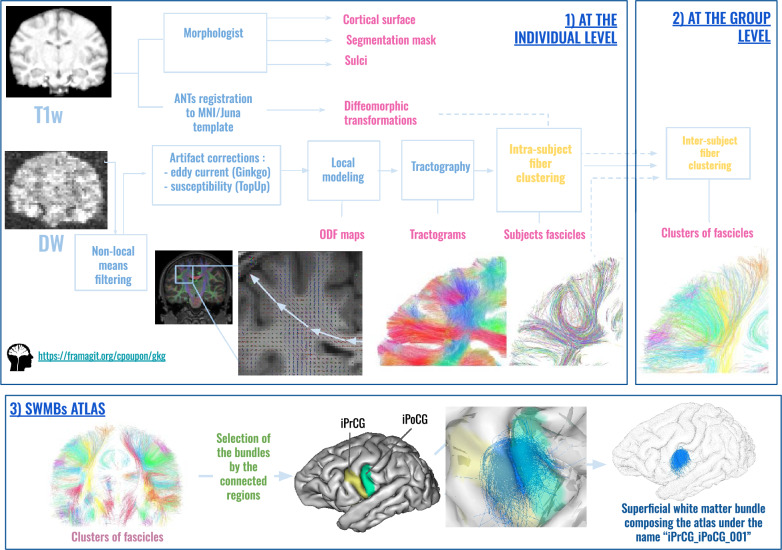
Pipeline of the different steps from raw anatomical and diffusion data to the species atlases. (1) At the individual level, anatomical MRI scans were processed using the *Morphologist* pipeline from Geffroy et al. (2011), enabling the extraction of various anatomical volumes and surfaces necessary for registration and subsequent analysis, such as cortical surface extraction, segmentation mask, and brain sulci. Diffeomorphic transformations to the MNI/Juna templates were computed based on the anatomical volumes. Diffusion MRI data underwent artifact correction, followed by the computation of orientation distribution functions, enabling deterministic tractography for each subject’s brain. Intra-subject fiber clustering was then applied to the tractograms to derive individual white matter fascicles. (2) At the group level, all individual white matter fascicles were aggregated, and a second inter-subject fascicle clustering was performed (using a normalized pairwise distance between the set of centroids representing the intra-subject fascicles) to identify clusters of fascicles representative of the group. (3) Atlasing the short white matter bundles (SWMBs): to atlas the short white matter bundles (SWMBs), clusters of fascicles containing short associative fibers were labeled according to the connected regions (e.g., "iPrCG" for "inferior Pre-Central Gyrus" and "iPoCG" for "inferior Post-Central Gyrus"). Pairing all clusters of fascicles facilitated the creation of a comprehensive atlas of superficial white matter bundles. Further details about this pipeline can be found in the "Fiber clustering" section. All processing steps were implemented using the CEA/NeuroSpin in-house C++ Ginkgo toolbox

Anatomical and diffusion MRI data were processed using a Python pipeline dedicated to the chimpanzee species developed with the CEA/NeuroSpin in-house C++ Ginkgo toolbox available at https://framagit.org/cpoupon/gkg and summarized in Fig. 1.

**Fig. 2:**
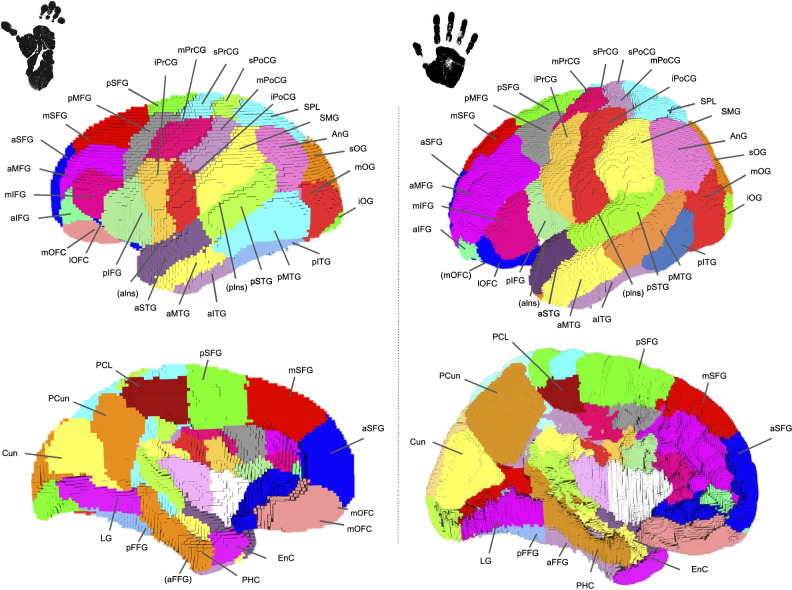
Cortical parcellation of the chimpanzee and human brains: (left) the chimpanzee cortical parcellation where the 38 left parcels are shown, these parcels were extracted manually from the DAVI130 atlas; (right) the human cortical parcellation used for the SWMB atlas, drawn on the MNI template using the Desikan-Killiany atlas as a reference for corresponding regions of the DAVI130. The 38 regions defined in Fig. 3 were manually drawn using *Voi viewer* from the Ginkgo toolbox

**Fig. 3:**
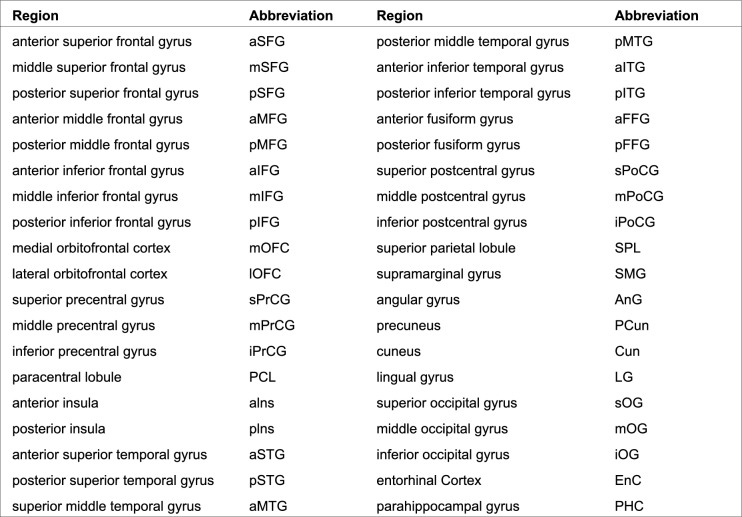
Table of corresponding cortical regions and labels used for the superficial atlases of the chimpanzee and human brains

All T1-weighted images of the 39 chimpanzees’ brains were matched to the Juna. Chimp chimpanzee template (template release: https://www.chimpanzeebrain.org/) (Vickery et al. 2020) using diffeomorphic direct and inverse non-linear 3D registration transformations computed using the ANTs software (Advanced Normalization Tools) (Avants et al. 2009). The Juna.Chimp template includes the DAVI130 cortical parcellation required for the computation of the SWMB atlas. In total, 76 regions corresponding to cortical areas from the template atlas were selected (38 per hemisphere) (see Figs. 2 and 3).

After correcting for the various dMRI imaging artifacts (Rician noise, eddy currents, susceptibility induced distortions), individual maps of local orientation distribution functions (ODF) were reconstructed from the multiple-shell diffusion-weighted 4D volume using the analytical Q-ball model (SH order = 6, $\lambda$ = 0.006) (Descoteaux et al. 2007). A whole-brain streamline regularized deterministic tractography algorithm (1 seed/voxel, forward step 0.4 mm, aperture angle 30$^{\circ }$, lower GFA threshold = 0.15) (Perrin et al. 2005) was then applied to each ODF map to generate streamlines within a propagation mask corresponding to the whole brain and established from the anatomical MRI, yielding the 39 chimpanzees’ individual tractograms composed of several millions of fibers.

Streamlines whose length did not belong to the 7–133 mm range were filtered out to target SWMB connectivity.

#### Humans

Similarly to chimpanzees, we designed an analysis pipeline for dMRI data processing based on the Ginkgo toolbox. Three consecutive steps were performed for each subject: (1) registration of the subject’s brain MRI to a common atlas space (the MNI ICBM 2009c non-linear asymmetric template) with the ANTS toolbox; (2) computation of the diffusion Orientation Distribution Functions (ODF) with the analytical Q-ball model; (3) computation of a whole-brain tractogram with a regularized deterministic algorithm.

To establish a relevant comparison between humans and chimpanzees, we carefully matched the post-processing pipelines and cohorts. Since the Desikan et al. (2006) and Davi130 atlases did not exactly present homologous cortical regions, we had to further refine the Desikan atlas and propose an inherited cortical atlas sharing the same number of cortical areas (76) as the chimpanzee DAVI 130 atlas (see Figs. 2 and 3). Refining the Desikan–Killiany atlas comprised the segmentation of some cortical areas such as: (1) the precentral and post-central gyrus, indeed the DAVI130 chimpanzee atlas included a subdivision of these two gyri into inferior, middle and superior Pre and Post-central gyrus (i.e "i,m,s Pr/PoCG") with borders corresponding to the superior and inferior borders of the hand-knob. The corresponding borders were used to subdivide the Desikan’s "i,m,s Pr/PoCG" cortical regions; (2) the same principle was used to subvdivide the Desikan’s "superior/ middle/inferior temporal" cortical areas into their "anterior/posterior - superior/ middle/inferior temporal" areas; following this labelling, each of the Desikan three temporal areas were divided into two subparts corresponding to new anterior and posterior temporal superior (aSTG/pSTG), temporal middle (aMTG/pMTG), and temporal inferior (aITG/pITG) areas, with a border between anterior and posterior areas corresponding anatomically to the intermediate "pli de passage" (Ochiai et al. 2004; (3) the "lateral occipital" label from the Desikan atlas was subdivided into "Superior Occipital Gyrus (sOG), Middle Occipital Gyrus (mOG), Inferior Occipital Gyrus (iOG)" as presented in the DAVI130; the sOG was separated by the mOG using the intra-occipital sulcus (as presented in Palejwala et al. (2020); the iOG was subdivided as corresponding to the most ventral part of the occipital lobe limited dorsally by the occipito-polar sulcus; (4) the "Superior frontal label" was subdivided into "anterior/middle/posterior superior frontal gyrus (a/m/pSFG)" regions matching the ones from DAVI130 with the border between the pSFG and mSFG corresponding to the projected limit between the rostral middle frontal and the caudal middle frontal Desikan labels; aSFG corresponds to the frontal pole. This refined Desikan-based human cortical atlas can be found at the following repository: https://doi.org/10.5281/zenodo.11174211.

### Fiber clustering

**Fig. 4:**
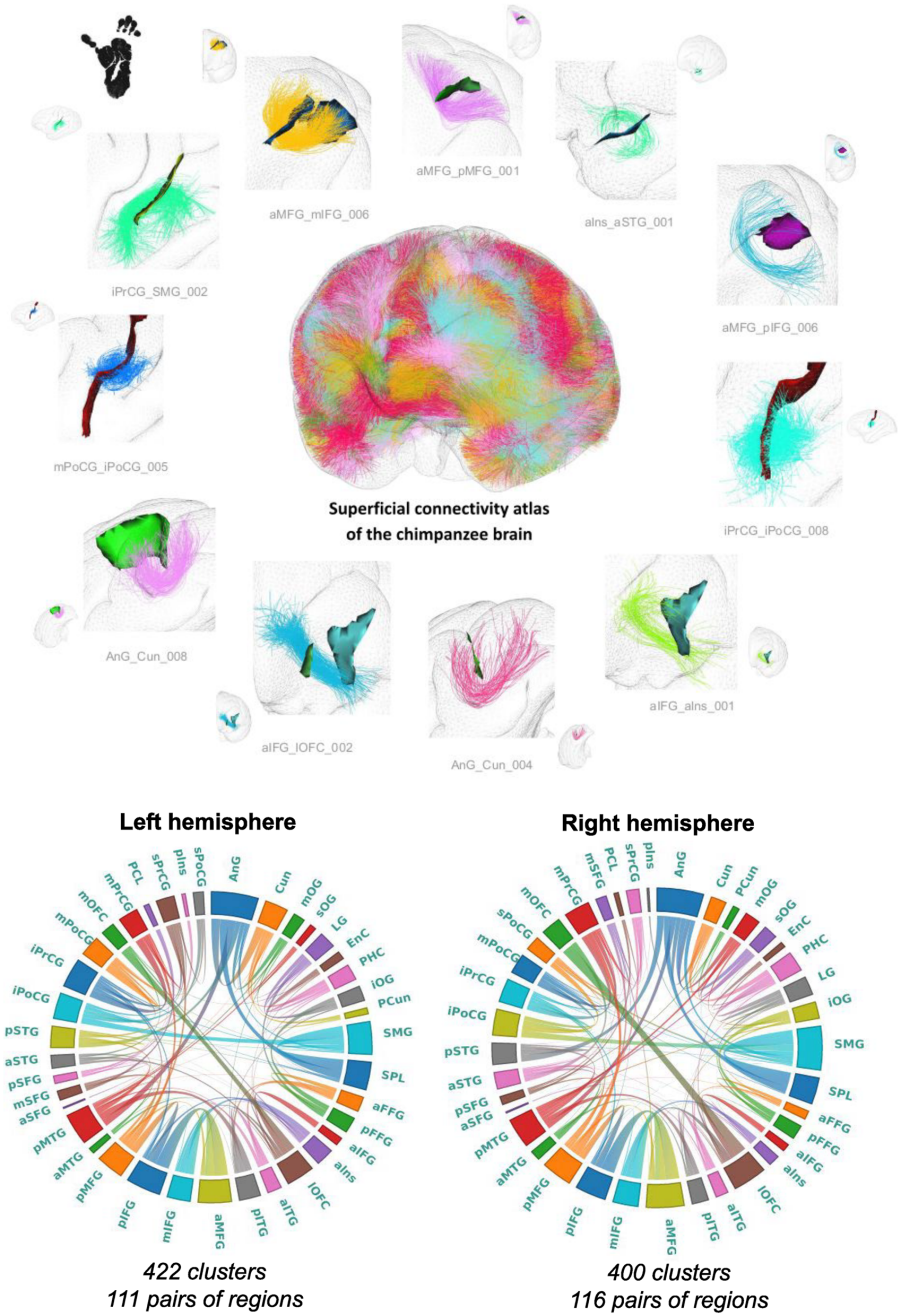
Superficial white matter fiber bundle atlas of the chimpanzee brain based on a fiber clustering from 39 subjects. Top: rendering of the superficial white matter atlas of the chimpanzee brain on the 3D pial surface, each bundle being represented with a different color. The atlas is surrounded by examples of superficial white matter bundles with the negative cast of nearby sulci. Bottom: left and right hemispheres connectivity matrices for bundles connecting the cortical regions, colors are related to the parcellation of the cortical mantle, thickness of regions proportional to the number of clusters

**Fig. 5:**
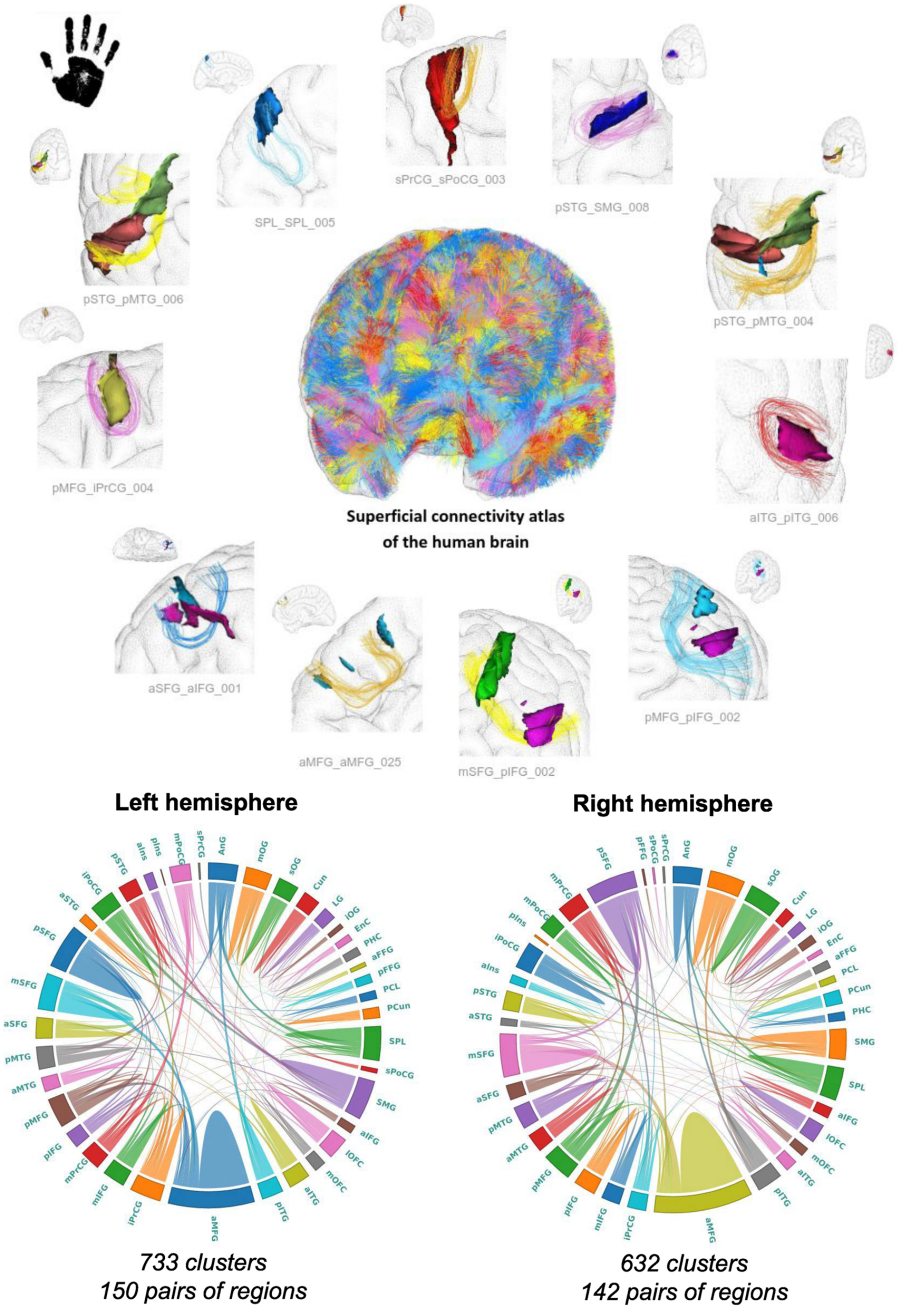
Superficial white matter fiber bundle atlas of the human brain based on a fiber clustering from 39 subjects. Top: rendering of the superficial white matter atlas of the human brain on the 3D pial surface, each bundle is represented with a different color. The atlas is surrounded by examples of superficial white matter bundles with the negative cast of nearby sulci. Bottom: left and right hemispheres connectivity matrices for bundles connecting the cortical regions, colors are related to the the parcellation of the cortical mantle, thickness of regions proportional to the number of clusters

In summary, the implemented analysis pipeline inherits the approach proposed in Guevara et al. (2011) and is further detailed in Chauvel et al. (2023):

- Subdivision of individual tractograms into gross fiber subsets (belonging to left and right hemispheres, inter-hemispheric region, brainstem and cerebellum), that are further subdivided into fiber lengths ranges, and projection of fibers into a density mask for each length range and region;
- Thresholding of each density mask to a binary mask corresponding to a specific gross region and length range;
- A first individual-level clustering: step 1) definition of parcels in the binary mask, corresponding to each range of fiber length stemming from the tractograms; step 2) computation of a connectivity matrix between all parcels measuring the number of fibers connecting two parcels (and being superior to a minimum number); step 3) hierarchical clustering of the connectivity matrix bringing out highly connected parcels forming "clusters"; step 4) each parcel cluster stems from a set of fibers making up a “fascicle”; step 5) the last step consists in a "watershed" aiming at further splitting fascicles with a fan shape using the localization of their fiber extremities.
- A second population-level clustering: step 1) diffeomorphic registration of each subject’s fascicles into a common template space (Juna template for chimpanzees, MNI space for humans); step 2) computation of fascicle clusters at the group level from the set of all fascicle centroids using a density-based spatial clustering algorithm (DBSCAN). This clustering is inferred from the affinity matrix of normalized symmetric mean of mean closest distances. Each resulting fascicle cluster represents a connection hub present in a large proportion of the subjects, putatively contributing to a target white matter bundle.
- Creation of SWMB atlases from the fascicle clusters using a semi-manual step. This last stage consists in aggregating for each target white matter bundle the set of fascicle clusters that matches the definition of the bundle. SWMB are identified and named by the pair of cortical areas they connect (see Fig. 2). It is of importance to note that a pair of cortical areas can be interconnected by more than one fiber bundle.
  On Figs. 4 and 5 are depicted global overviews of the SWMB atlases of the human and chimpanzee brains, with the circular matrix representing the existing connections between cortical regions, for both left and right hemispheres. On the Appendix are are presented the two tables of the corresponding superficial bundles from the SWMB atlas, with the corresponding number of clusters found in Figs. 4 and 5.

### Empirical exploration of the superficial bundles

**Fig. 6:**
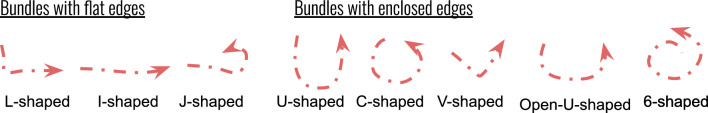
Schematic representations of the different shapes that could be identified for the SWMB atlases. Two major morphologies are found, composed by flat and enclosed edges, and among them we distinguished: L-shaped, I-shaped, J-shaped, U-shaped, C-shaped, V-shaped, Open-U shaped and 6-shaped morphologies

Prior investigations of the SWMBs in humans have unveiled a limited repertoire of characteristic shapes defining these bundles, an observation previously documented (Labra Avila 2020). Being relatively understudied structures, SWMBs exhibit shapes that remain poorly understood and appear to be strongly related to the underlying sulco-gyral patterns. The central question at hand is whether the diversity of SWMB shapes observed in humans is also mirrored in chimpanzees (Fig. 6).

In the context of this research, an initial empirical approach was employed to identify the various relevant shapes of SWMBs. This method entailed a visual examination and classification of hundreds of bundles composing the atlas based on their shapes. The *Anatomist* viewer tool (Rivière et al. 2022) allowing to render simultaneously any SWMB across subjects facilitated this meticulous and time-intensive process performed by two independent experts. The results are presented in the Appendix, A2 section for the left and right hemispheres of the human brain, as well as for both of these regions of the chimpanzee brain. Specific morphological attributes, including flatness, curvature, and elongation of the bundle, served as criteria for the comprehensive classification of all observed bundles independently of their actual length.

### Geometric morphological approach

**Fig. 7:**
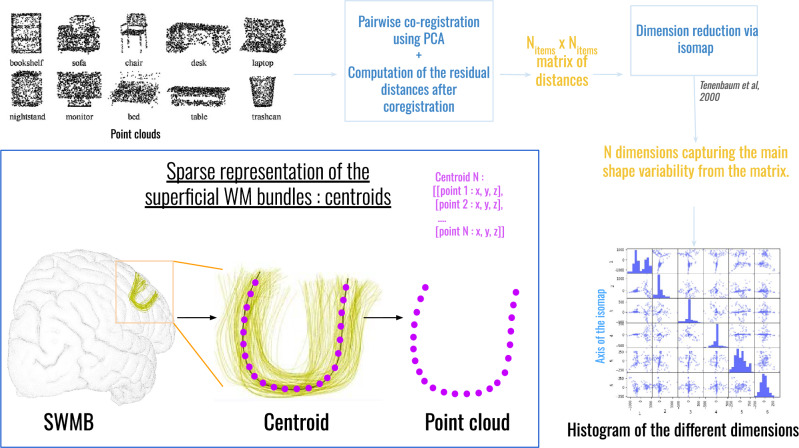
Pipeline of the different steps from point clouds to the description of shape variability through an isomap algorithm

To attempt to transcend the limitations associated with empirical methods that are prone to subjective biases, we implemented an automated shape classification pipeline based on the isomap algorithm. This approach, drawing inspiration from the work from Sun et al. (2017), relies on the description of each geometric object to be classified using point clouds (see Fig. 7). By employing point clouds as inputs, this methodology aims to depict and encapsulate the variations in shape across different entities. This is achieved by computing the distances between points and subsequently reducing the dimensionality, as outlined in Tenenbaum et al. (2000).

As a result of this analysis, a collection of axes within the newly defined n-dimensional space aids in translating the morphological variability of shapes. We therefore performed a clustering algorithm on this result to identify the different average shapes and their localisation.

In both the human and chimpanzee brains, the representation of the geometry of the superficial fiber bundles underwent a simplification process, wherein a single representative fiber, referred to as the ’centroid’ fiber, was derived using the Gingko toolbox (see Fig. 7). This sparser representation of SWMBs resulted in a straightforward point cloud description for each SWMB. This point cloud representation serves as an efficient means to analyze shape variations among SWMBs while retaining the key characteristics of their shapes.

Before clustering SWMB shapes using the isomap algorithm, their point cloud representations were aligned into a common space using a Principal Component Analysis (PCA) approach. This initial step involved extracting the three primary axes of each point cloud and aligning these principal directions across all point clouds through the application of optimal affine transformation matrices (Bellekens et al. 2014). While more intricate alignment strategies involving point clouds, such as singular value decomposition(SVD)-based and Iterative Closest Point-based (ICP) strategies were explored both individually and following the initial PCA step, no significant difference in results were observed that would justify the increased computational complexity compared to the PCA approach.

The geometrical distance between two fibers A and B was calculated using the symmetric point to point pairwise distance between $C_1$ and $C_2$, two fibers represented by $N_p$ control points $\left\{ P_{c_1}(i)\right\}$ and $\left\{ P_{c_2}(i)\right\}$:

1 $$\begin{aligned}&d_{pairwise}(c_1,c_2) \nonumber = min \left( \sqrt{\sum _{i=0}^{N_p-1} (P_{c_1}(i) - P_{c_2}(i))^2}, \right. \nonumber \left. \sqrt{\sum _{i=0}^{N_p-1} (P_{c_1}(i) - P_{c_2}(N_p-i))^2}\right) \end{aligned}$$

All the distances between all centroids were stored in a $N_p$ x $N_p$ symmetric matrix.

Our clustering strategy was twofold: first, the isomap algorithm was applied to reduce the N-dimensional space as described in Tenenbaum et al. (2000). Its number-of-neighbors parameter was empirically chosen at a value lower than 5. We observed that a reduction to only 2 dimensions effectively retained sufficient information for subsequent clustering steps. Keeping higher dimensions did not practically bring relevant information to their different bundle shapes. Next, we proceeded with the identification of clusters within the isomap-reduced space (embedding) using a k-means algorithm. The selection of the appropriate number of clusters, denoted as ’k’, was also guided by empirical assessment of the results. The value of ’k’ was progressively increased until the resulting clusters distinctly exhibit different shapes. Subsequently, we aligned the point clouds of all centroids to the reference point cloud corresponding to the central cluster. We then computed a new average point cloud from all the aligned point clouds to represent the general shape characteristics of the fibers within the cluster.

The clustering pipeline employed in this study has been implemented and is available as a Python module, as detailed in Pascucci et al. (2022).

This methodology was applied to the chimpanzee and human SWMB datasets in a similar way.

## Results

### Empirical exploration of the chimpanzee superficial connectivity

The empirical classification already provided valuable insights into the anticipated patterns of shape clustering, assessing the presence of various shapes within the SWMBs, including U-fibers and V-fibers. Furthermore, a noticeable symmetry has been observed in the distribution of these shapes between the two cerebral hemispheres.

**Fig. 8:**
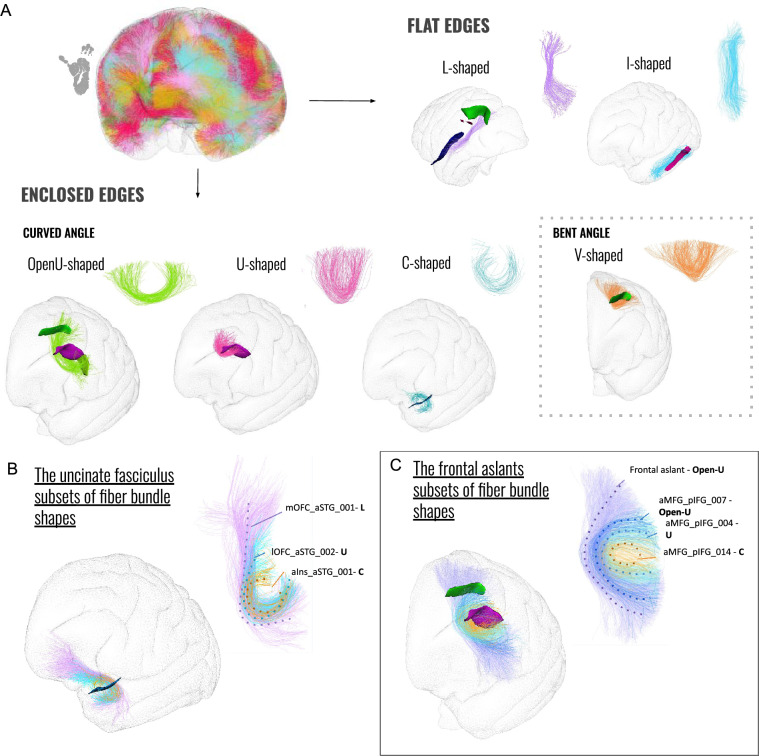
Superficial white matter bundle shapes of the chimpanzee brain. **A** Fiber bundles are categorized into various groups based on their shapes, ranging from bundles with flat edges to those with enclosed edges. Each group is supported by the main fiber shape and an example related to crossed sulci projected onto the Juna.Chimp template chimpanzee pial surface is given. **B and C** Instances of well-recognized bundles, along with their subdivisions and corresponding fiber bundle morphologies, are provided. Specifically, the uncinate fasciculus and the frontal aslants are presented

**Fig. 9:**
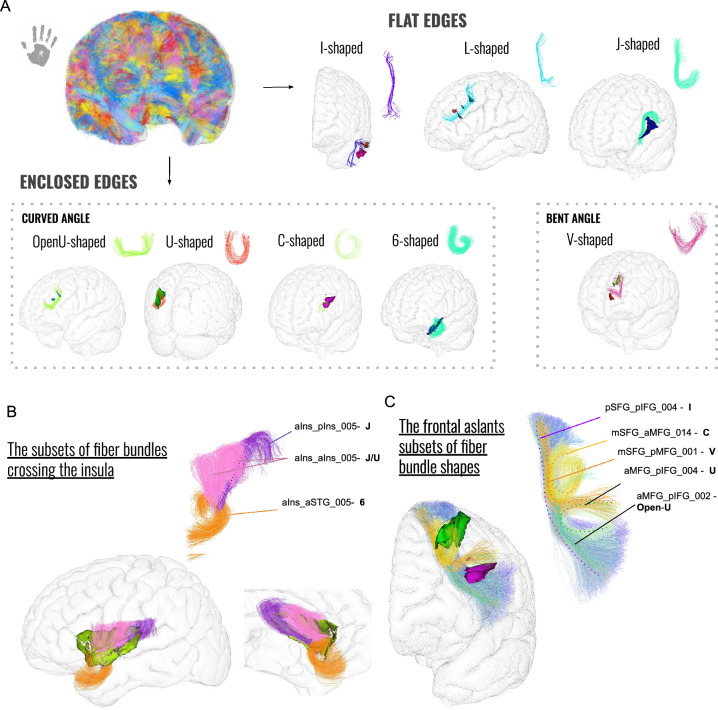
Superficial white matter bundle shapes of the human brain. **A** Fiber bundles are categorized into various groups based on their shapes, ranging from bundles with flat edges to those with enclosed edges. Each group is supported by the main fiber shape and an example related to crossed sulci projected onto the MNI template human pial surface is given. **B** Particular instances of fiber bundle shapes crossing the insula. **C** The frontal aslants along with the bundle subdivisions and corresponding identified shapes

Most of the SWMBs of the chimpanzee brain atlas could be classified into six fine categories representative of their shapes, as depicted in Fig. 8A. On a coarse scale, two primary superficial bundle shapes were observed depending on the degree of fiber aperture including: (1) enclosed-edge bundles and (2) flat-edge bundles (Fig. 9).

**Fig. 10:**
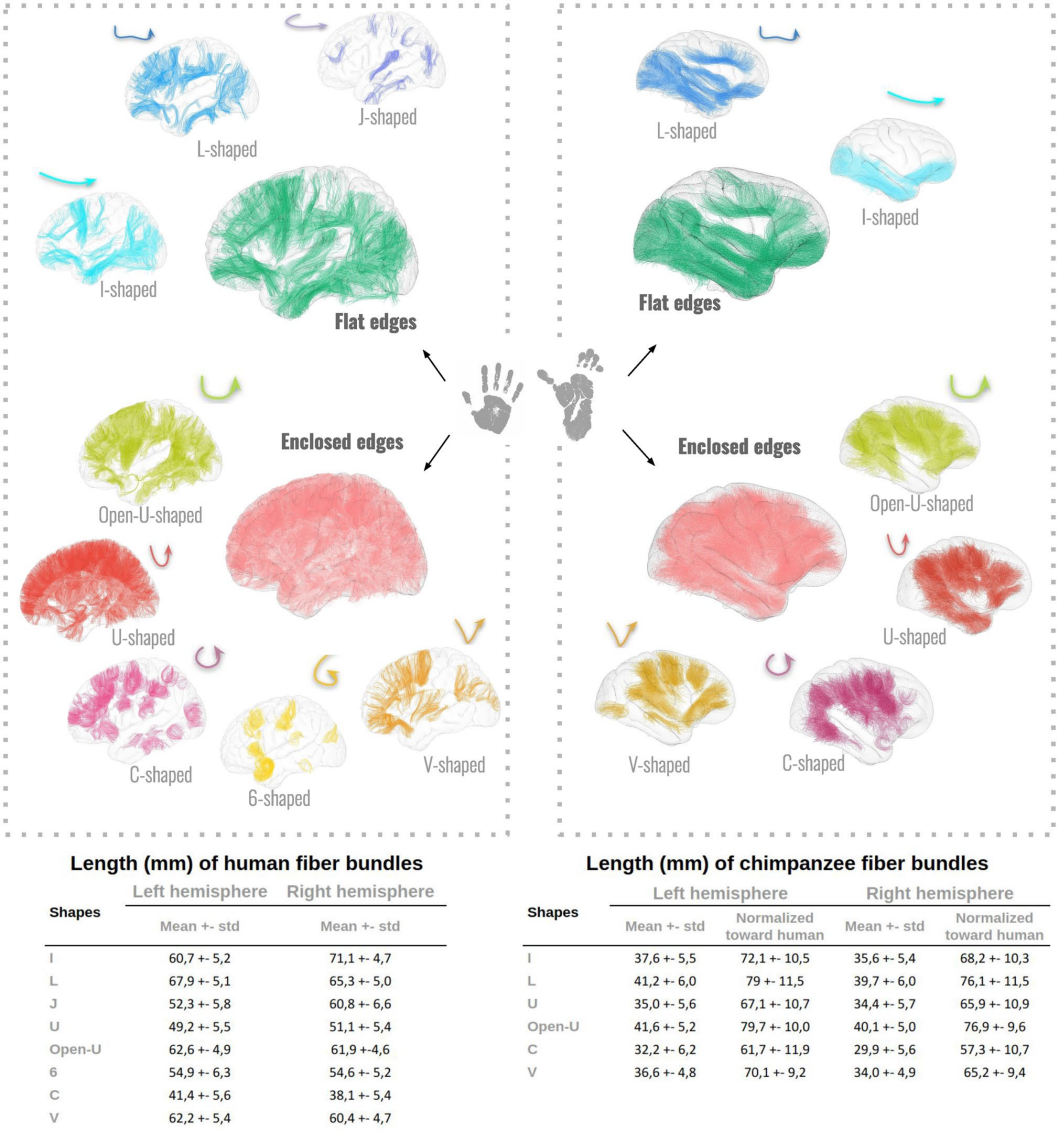
(Top) Fiber bundle shapes empirically seen in the human (left) and chimpanzee (right) brains, accompanied by synthetic shape representations. (Bottom) Table of correspondence between the different observed shapes and related fiber bundle lengths (mean ± standard deviation) for the left and right hemispheres, in both species. Mean lengths of chimpanzee fiber bundles normalized by the cortical surfaces toward human are displayed in the columns entitled "normalized toward human". Chimpanzee bundle lengths have been normalized to human bundle lengths using a multiplicative factor based on the square root of the ratio between the pial surface areas of the two species

**Fig. 11:**
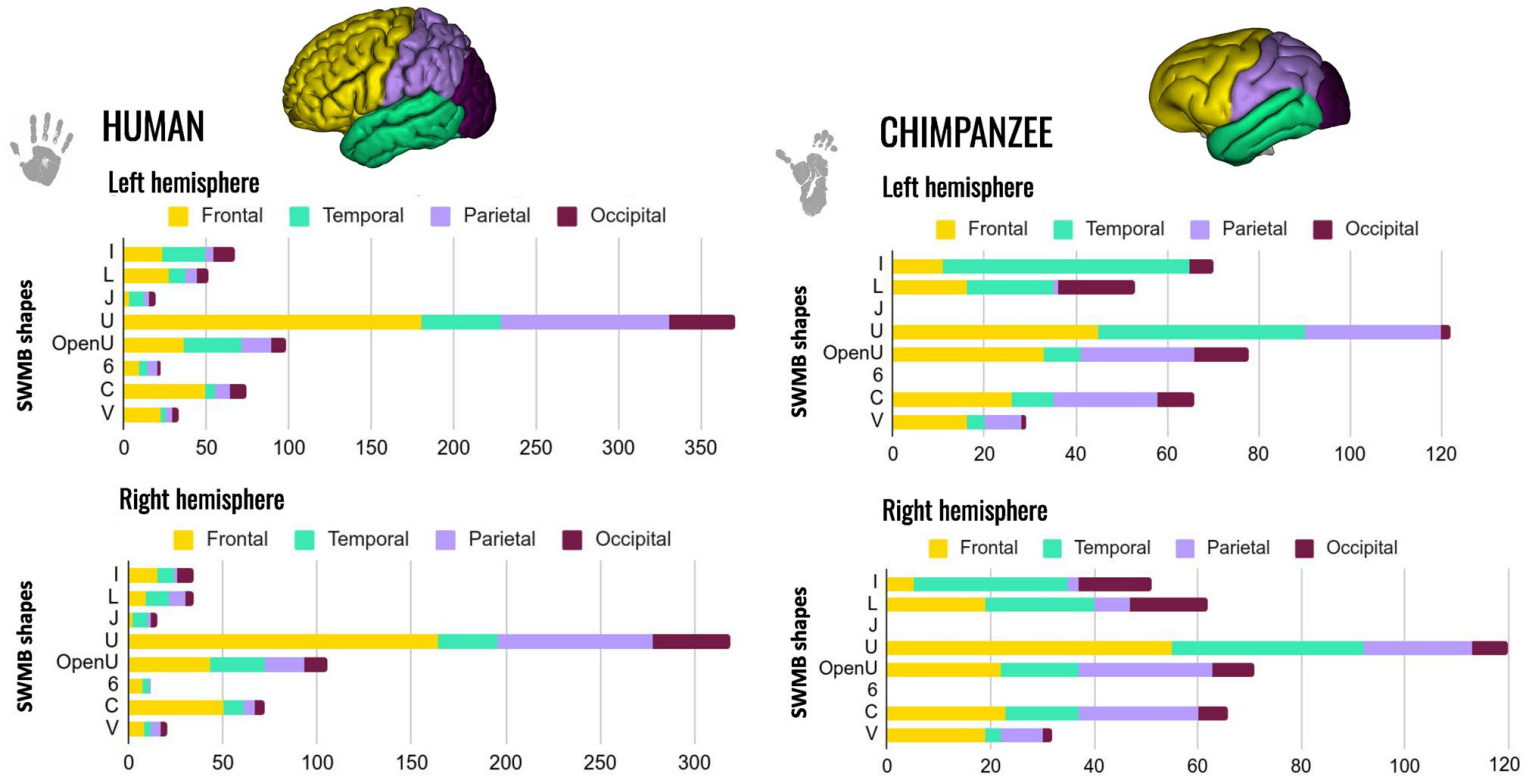
Histograms of superficial white matter bundles shape and their number in brain lobes (frontal, temporal, parietal and occipital) of the left and right hemispheres for humans (left) and chimpanzees (right)

While enclosed-edge bundles appeared to be distributed throughout all brain lobes, as illustrated in Figs. 10 and 11, bundles with I and L-shaped configurations were primarily confined to the ventral region of the brain. However, some sections of the corpus callosum, which were considered superficial bundles, exhibited a flat shape in the superior medial portion of the cerebral hemispheres, running adjacent to the cingulum. In addition to flat-edge bundles, enclosed-edge bundles could be further subdivided into two primary subgroups: (1) bent-angled fiber bundles and (2) curved fiber bundles. Notably, these two categories, which are not uniformly distributed in the brain (see Fig. 11), exhibited distinct morphological characteristics. The bent-angled bundles correspond to V-shaped bundles, resembling those observed in superficial bundles in humans, as documented in Labra Avila (2020). A careful examination of these V-shaped bundles revealed that the most angular ones tend to be situated in the frontal and temporal lobes and represent a minority within the brain’s overall composition (refer to Fig. 10). The tips of these V-shaped bundles occasionally exhibited a fan distribution of bundles that connect the cortical ribbon.

Within the category of curved bundles, various types of bundles were observed and grouped into three subcategories based on the degree of fiber edge enclosure. The first subgroup consisted of C-shaped bundles, named so due to their fully enclosed extremities directed toward the bundle’s center of gravity, resembling the letter "C". These bundles are relatively scarce within the brain but are distributed across all brain lobes. Moreover, they are characterized by having the shortest fiber length among the bundle types. The second subgroup comprises the majority of bundles (see Fig. 11) and is referred to as "U-shaped bundles" due to their resemblance to the letter "U". These bundles possess enclosed extremities, although they are less sharply bent compared to C-shaped bundles. They can be categorized among the medium length bundle types.

The third and final subgroup is composed of "Open-U" bundles, which exhibited a configuration resembling the letter "U", albeit with more open extremities compared to the previous subgroup. These bundles are the longest in terms of fiber length (refer to the table in Fig. 10). Distinguishing between Open-U bundles and V bundles could sometimes be challenging due to their similar shapes.

The specific case of the frontal aslants is illustrated in Fig. 8C. since they aggregate different shapes from superficial to deep location. While they are commonly categorized as deep white matter bundles in the literature, it is not inaccurate to consider them as superficial, since they include cortico-cortical fibers with relatively short lengths. We therefore retained them within the SWMB atlas, consequently leading to a significant segmentation of their subcomponents as determined by the fiber clustering algorithm. These subcomponents serve as noteworthy examples of the continuous variability in shape that is observed as one progresses from subcortical to deeper brain regions. A similar situation concerns the uncinate fasciculus, as depicted in Fig. 8B. A fiber clustering within the uncinate fasciculus revealed the presence of at least three subcomponents connecting distinct cortical regions, each seemingly associated with distinct shape characteristics.

### Empirical comparison with the human superficial connectivity

A similar empirical study of the human brain superficial connectivity yielded bundle shapes similar to that of the chimpanzee brain (see Figs. 9, A. and 10) including the U-shaped fiber bundles, C-shaped fiber bundles, I-shaped fiber bundles, L-shaped fiber bundles and V-shaped fiber bundles. However, some different shapes emerged in humans like the "6-shaped" and "J-shaped" fiber bundles.

Interestingly, most of the fiber bundles composing the human specific 6 and J shapes correspond to fiber bundles crossing the insula in humans as it can be seen in Fig. 9B. Fiber bundles composing the J shape connect the inferior frontal cortex and spread along riding the insula from the inferior frontal gyrus to the temporo parietal junction, reaching more lateral frontal cortical regions. Thick fiber bundles depicting the 6 shape also connect the insula from the same hub as the J-shaped fiber bundles. However, instead of running caudally, they twist medially and ventraly to reach the temporal pole.

Figure 9 visually illustrates that in comparison to the chimpanzee brain, the human frontal aslant exhibits greater thickness and comprises a higher number of subparts. Remarkably, both the chimpanzee and human frontal aslants display a diverse range of fiber bundle shapes, including V, I, C, U, and Open-U, as depicted in Fig. 9C.

A high degree of correlation was observed between the lengths of various fiber bundles observed in both human and chimpanzee brains and their corresponding shapes as shown in Fig. 10. Notably, in both species, the flattest fiber bundles, denoted as I and L-shaped, are the longest ones, with respective average lengths of approximately 36.6 mm (I)/40.5 mm (L) for chimpanzees and 65.5 mm (I)/66.6 mm (L) for humans, as depicted in Fig. 10. Interestingly, Open-U and V-shaped fiber bundles also demonstrated notable lengths, measuring on average approximately 40.9 mm (Open-U)/35.3 mm (U) in chimpanzees and 62.3 mm (Open-U)/61.3 mm (U) in humans. In contrast, the smallest fiber bundles in both species belong to the C-shaped group, with average lengths (combining both hemispheres) of around 35.3 mm for chimpanzees and 61.3 mm for humans. Interestingly, regarding the normalized values of WM bundle lengths computed for chimpanzee, scaled with respect to the ratio between human and chimpanzee brain cortical (pial) surfaces, white matter bundles are globally longer in chimpanzees than in humans, all shape categories taken together. Indeed, normalized values for the chimpanzee left hemisphere revealed higher values of lengths by 19% for I-shaped WM fiber bundles, 16% for L-shaped WM fiber bundles, 36% for U-shaped WM fiber bundles, 27% for Open-U-shaped WM fiber bundles, 49% for C-shaped WM fiber bundles and 13% for V-shaped WM fiber bundles. Normalized values for the chimpanzee right hemisphere revealed higher values of lengths by 16% for L-shaped WM fiber bundles, 29% for U-shaped WM fiber bundles, 24% for Open-U-shaped WM fiber bundles, 50% for C-shaped WM fiber bundles and 8% for V-shaped WM fiber bundles. The only exception was found for I-shaped WM fiber bundles where WM fiber bundles of the right hemisphere were shorter for the chimpanzee compared to human by 4%.

### Isomap-based classification

**Fig. 12:**
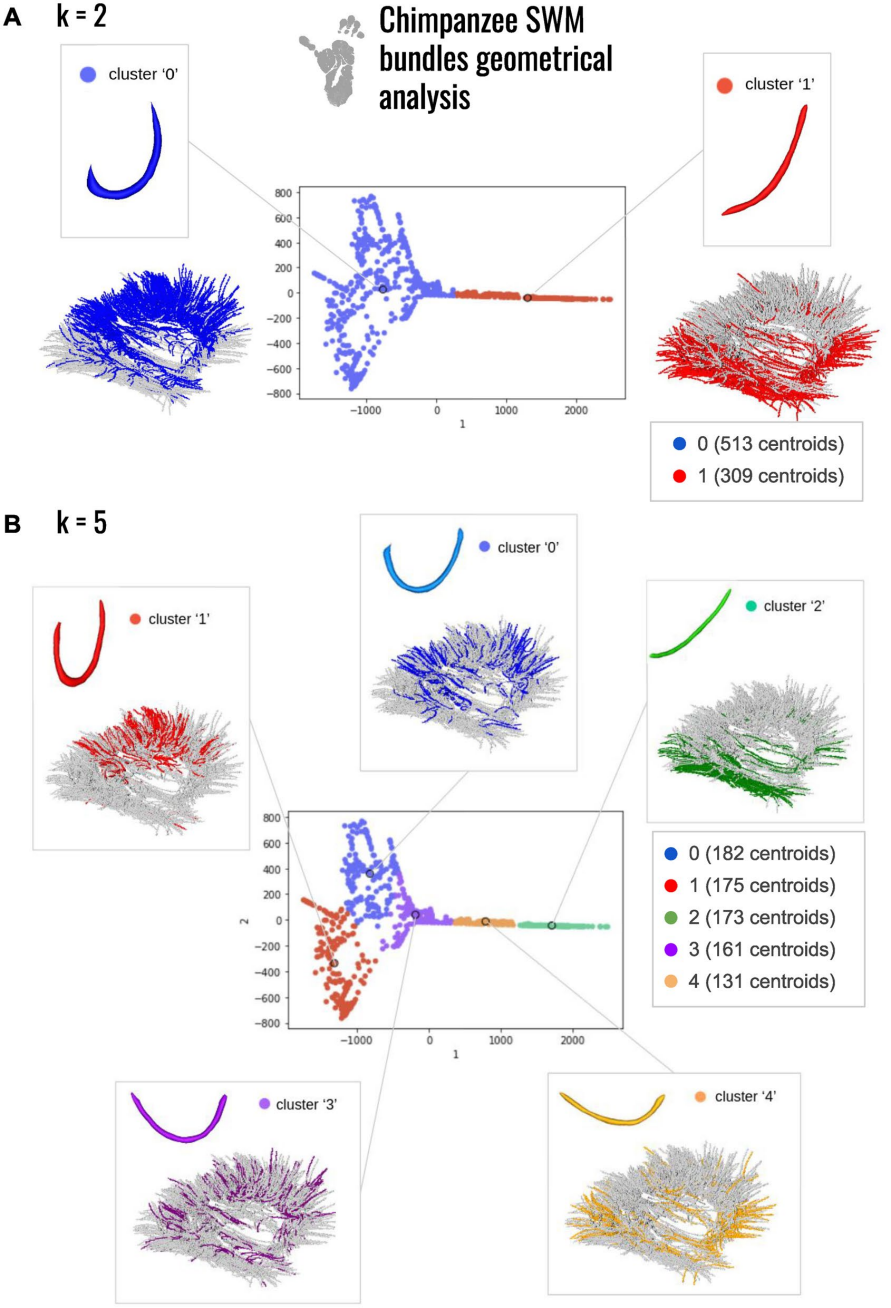
Morphological investigation of the superficial white matter bundles of the chimpanzee brain. **A** 2 clusters of fiber bundles are identified with average shapes (k = 2) and related number of fiber bundles belonging to each shape. **B** 5 clusters of fiber bundles are identified with average shapes (k = 5), number of neighbors = 5, and related number of fiber bundles belonging to each shape

**Fig. 13:**
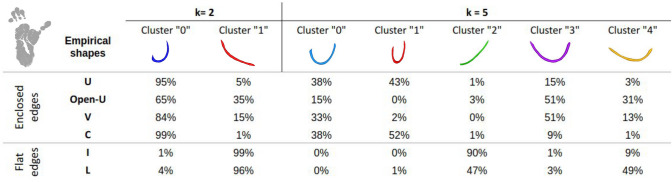
Percentage of white matter bundles belonging to empirically defined (U, Open-U, V, C, I, L) shapes contributing to each cluster of a chimpanzee isomap-based classification for k = 2 and k = 5 respectively

**Fig. 14:**
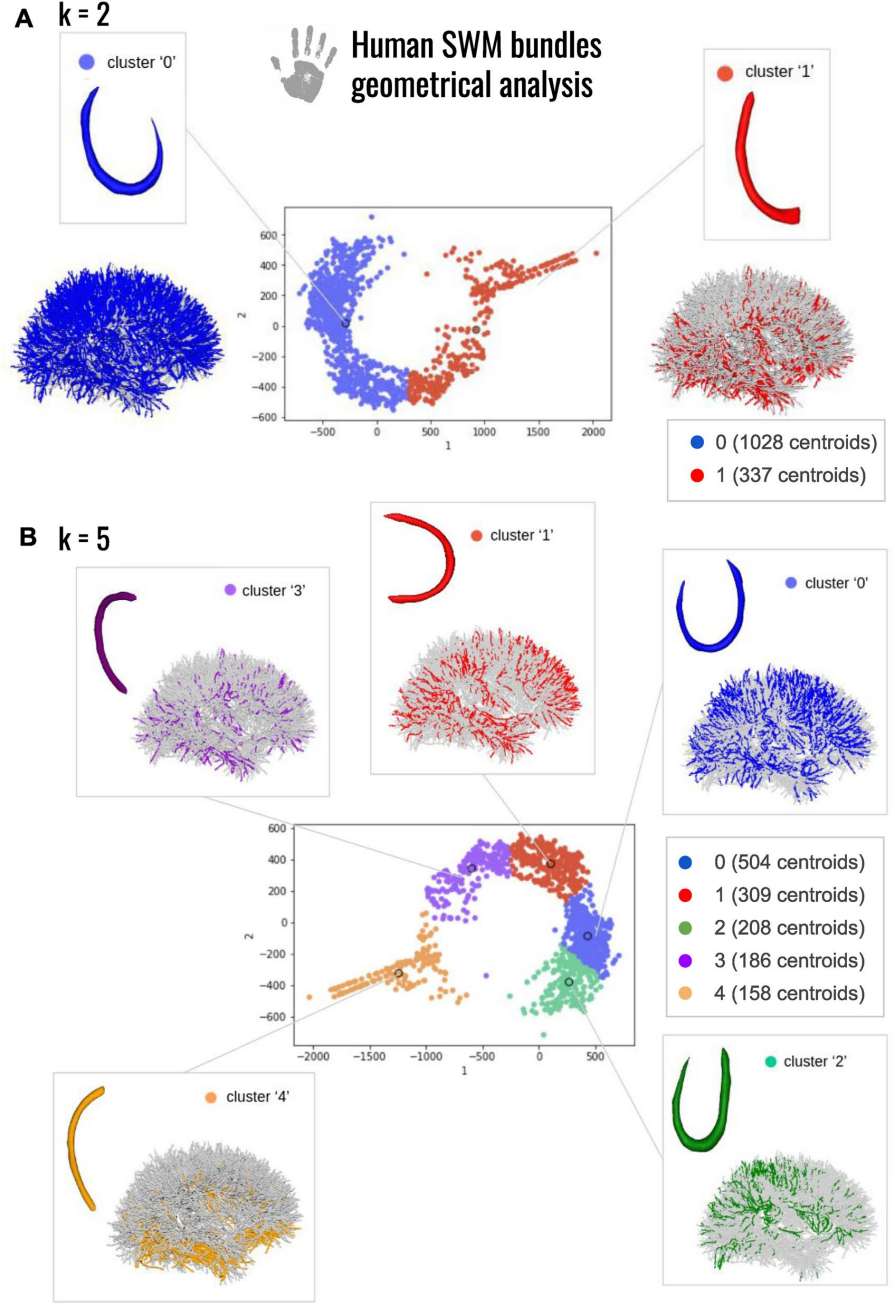
Morphological investigation of the superficial white matter bundles of the human brain. **A** 2 clusters of fiber bundles are identified with average shapes (k = 2) and related number of fiber bundles belonging to each shape. **B** 5 clusters of fiber bundles are identified with average shapes (k = 5), number of neighbors = 5, and related number of fiber bundles belonging to each shape

While the empirical study already shed the light on specificities of the human superficial connectivity with respect to the chimpanzee connectivity, the application of the isomap-based classification method to SWMBs allowed us to more objectively corroborate these observations, eliminating potential biases associated with human visual inspection. To achieve this, we explored the main variability of the bundle shapes with a low dimensional space as described in Tenenbaum et al. (2000). In our study, setting the ’k’ parameter to 5 yielded the best number of clusters exhibiting significantly distinct shapes (Figs. 12, 13, 14), as shown in Figs. 12B and 14B.

**Fig. 15:**
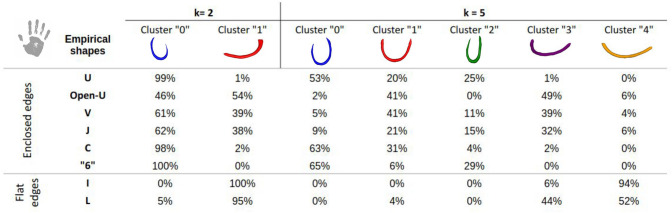
Percentage of white matter bundles belonging to empirically defined (U, Open-U, V, J, C, 6, I, L) shapes contributing to each cluster of a human isomap-based classification for k= 2 and k= 5 respectively

As illustrated in Figs. 12 and 14, the isomap analysis resulting from k = 2 revealed the emergence of two primary shapes, one corresponding to SWMB displaying enclosed extremities, and the other corresponding to SWMB exhibiting open extremities (flat). In both species, flat bundles were predominantly located in the ventral region of the brain, while curved fiber bundles were primarily distributed in the middle-dorsal region. This observation confirmed previous empirical findings (see Figs. 13 and 15). Notably, in humans, curved fiber bundles are more widely distributed throughout the brain compared to chimpanzees, where they appear to be largely restricted to the dorsal part of the brain.

Pushing further the number of clusters to k = 5, the isomap clustering revealed further distinct patterns in the closure of fiber bundle shapes between the chimpanzee and human brains (i.e if the fiber extremities are more or less closed, as an “O” or straight as an “I”). In chimpanzees, fiber bundle shapes evolve from flat shapes ventrally to more closed shapes dorsally. However, in humans, bundle shapes tend to close from the ventral anterior region to the dorsal caudal part of the brain. While bundle shapes appear relatively similar between human and chimpanzee brains, human bundles display greater enclosure of their extremities and exhibit different spatial distributions over the cortex.

At a finer scale, in the chimpanzee brain, the algorithm identified specific clusters of shapes with specific distribution of these patterns in the brain. For instance, within cluster corresponding to label "0" containing empirically defined U-, C- and V-shaped WM bundles (see Fig. 12B. and corresponding table of comparison between the isomap and the empirical classifications Fig. 13), most WM bundles are concentrated in the frontal and parietal lobes, less present in the superior temporal lobe and limited in the anterior temporal lobe. The cluster corresponding to label "1", depicting a U-shape containing empirically defined U-shape and C-shape fiber bundles, is primarily located in the superior frontal and parietal lobes, minimally present in other lobes. The cluster corresponding to label "2", representing the flattest shapes, predominantly occupies the lower-ventral part of the temporal lobe. The cluster corresponding to label "3", composed of a mixture of Open-U and V-shaped bundles, is distributed across all lobes, but mostly in the frontal and parietal lobes and fewer in the superior gyri of the temporal lobe. Lastly, the cluster corresponding to label "4" populated by curved fiber bundles mostly corresponding to empirically defined Open-U and L shapes is primarily found in the inferior frontal and occipital lobes.

In the human brain, U-shaped fiber bundles with enclosed extremities are uniformly distributed across all lobes. In contrast, flat fiber bundles are mainly located in the ventral part of the brain, a characteristic shared with chimpanzees (see Fig. 14A).

Further analysis of classification into five clusters (Fig. 14B) revealed slight differences in shapes between humans and chimpanzees, see also Fig. 15.

Different types of enclosed WM bundles emerged: the cluster corresponding to label "0" and the cluster corresponding to label "2". As these clusters both resembled U-shaped WM fiber bundles, the cluster corresponding to label "2" is composed of WM bundles presenting a wider opening, while cluster labelled "0" embeds WM shapes whose closer extremities resemble the "C" and "6" shapes. WM fiber bundles composing cluster labelled "0" were distributed throughout all brain lobes. Cluster labelled "1", displaying an Open-U shape and containing mostly empirically defined Open-U and V-shaped WM bundles, was present in all lobes except around the central sulcus for which WM bundles connecting the pre-central and post-central gyri were associated to clusters "0" and "2". Cluster labelled "2" composed of enclosed shapes was primarily located in the parietal and occipital lobes, to a lesser extent in the frontal lobe and almost never in the temporal lobe. WM fiber bundles composing cluster labelled "3," depicting an Open-U morphology with a greater aperture angle than in cluster labelled "1", due to the presence of empirically classified L-shaped additionally to Open-U-shaped fiber bundles, were predominantly found in the ventral part of the brain, particularly in the inferior temporal lobe. Some bundles were also present in the frontal, parietal, and occipital lobes, in their ventral portions. The last cluster, labelled "4", representing flat shapes, was mainly concentrated in the inferior frontal and temporal lobes, and more generally populating the ventral part of the brain in comparison to cluster labelled "3".

**Fig. 16:**
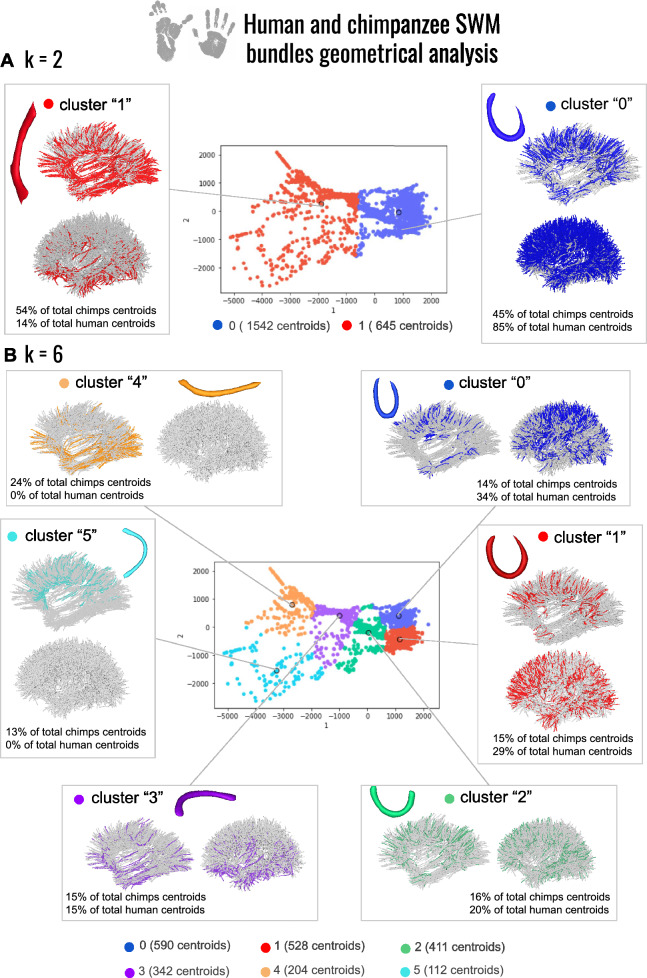
Morphological investigation of the superficial white matter bundles in chimpanzee and human brains. **A** 2 clusters of fiber bundles are identified with average shapes (k = 2). **B** 6 clusters of fiber bundles are identified with average shapes (k = 6). The related number of fiber bundles belonging to each shape in total is displayed. Under each cluster, the percentage of centroids belonging to each average shape for each species is indicated

**Fig. 17:**
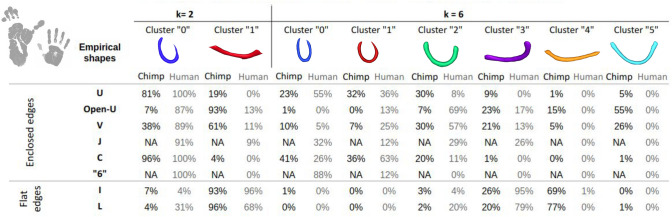
Percentage of white matter bundles belonging to empirically defined (U, Open-U, V, C, I, L) shapes contributing to each cluster of a joint human-chimpanzee isomap-based classification for k = 2 and k = 6 respectively

To further compare the variability in average shapes between both species, a new isomap analysis was launched with the two species SWMB centroids mixed together, see Fig. 16. All bundles are normalized by default using PCA-based analyses. The corresponding table providing the correspondence between the isomap-based and the empirical classifications is shown in Fig. 17.

Observations from Figs. 16A and Fig. 17 revealed that, consistent with findings in the specific isomap spaces of both species (see Figs. 12 and 14) the predominant fiber bundle shapes are the curved shape (cluster "0") and straight/flat shape (cluster "1") for the two species. Curved fiber bundles are predominantly located in the dorsal part of the chimpanzee brain and are more widespread in the human brain, aligning with previous observations done at the individual species level.

The flattest fiber bundles are primarily located in the ventral part of the human brain, although they appear to be more widespread in chimpanzees. It is noteworthy that combining data from both species emphasizes a higher proportion of curved fiber bundles in humans (accounting for approximately 85% of their total number of fiber bundles) compared to chimpanzees (45%). This underscores a notable contrast in the overall degree of curvature of fiber bundles between the two species.

An analysis of the clustering results using a target of six clusters showed that we do find similar patterns to those identified for each species: cluster corresponding to label "0" is composed of WM bundles whose mean shape depicts tightly enclosed edges actually corresponding to WM bundles empirically defined as U-, C- or "6-" shaped; similarly, the second cluster with label "1" mostly contains empirically defined C-shaped WM bundles; the third cluster with label "2" mostly contains empirically defined U-, Open-U- and V-shaped WM bundles; the fourth cluster corresponding to label "3" contains straighter shapes corresponding to empirically defined J-, I- and L-shaped WM fiber bundles. The strategy used for the geometric analysis was to increase progressively the value of the number of clusters as long as the resulting clusters distinctly exhibited diverse shapes. In the case of the analysis mixing both humans and chimpanzee data (Fig. 16), the value of ’k’, defining the number of clusters could be increased to six, depicting six different fiber bundle shapes. Intriguingly, two average fiber bundle shapes corresponding to clusters "4" and "5" emerged which belong exclusively to chimpanzees (concerning 24% and 13% for chimpanzees respectively for clusters "4" and "5", and 0% in humans for both). The averaged shape linked to these clusters appeared as straight/flat for cluster "4" and Open-U for cluster "5", with more open extremities than in the case of cluster "2".

A comprehensive illustration of the identified average shapes, along with the corresponding bundle count associated to each shape is provided in the Appendix section (A3) for various k values.

## Discussion

This study represents a novel investigation into the morphological organization of SWMBs in both the chimpanzee and human brains, aiming to compare their organizational and morphological similarities or differences. The challenge in addressing this question lies in its inherent subjectivity, which we sought to mitigate by complementing empirical observations with a geometric analysis designed to classify SWMB shapes in an automated and reproducible manner.

Our approach involved empirical observations combined with an algorithmic categorization of bundle shapes, aiming to highlight bundle shape differences within and between the two species, shedding light on their cortical connectivity and their potential evolution in relation to newly acquired cognitive functions.

If no prior studies have reported differences related to the link between sulco-gyral patterns and underlying white matter connectivity between humans and chimpanzees, several hypotheses can be proposed. In this study, at the individual scale, both human and chimpanzee species depicted various bundle shapes, from bundles displaying enclosed extremities to others having a flat morphology. However, when considering both species collectively, particularly through morphological analysis, a noticeable distinction emerged: chimpanzees exhibited generally flatter and longer SWMBs compared to humans. This morphological difference was especially significant in the ventral part of the brain, encompassing the inferior temporal lobe, inferior occipital lobe, and a portion of the inferior frontal lobe. This difference may be attributed to gyrification differences. Indeed, studies have shown that the gyrification index, a measure of cortical folding complexity, is typically greater in humans compared to chimpanzees (Rogers et al. 2010; Zilles et al. 1989). Indeed, the number of sulci in humans is higher, and their depth is also greater, suggesting that more short association fiber bundles are likely present in humans. This is supported by the findings of this study, which identified 40% more SWMBs in humans compared to chimpanzees. With the increase in size of the human brain over the course of evolution and split from chimpanzees, there has been the development, rearrangement, and enlargement of the brain, with major increase in frontal (Passingham and Smaers 2014) and parietal areas (Goldring and Krubitzer 2020). This led to increased folding of the cortical mantel, resulting in the emergence of deeper sulci. Indeed, during typical neurodevelopment, the first set of folds, known as primary sulci and gyri, begins to appear as the brain expands and the cortex thickens. These primary folds occur in specific regions of the brain (central sulcus, sylvian fissure, parieto-occipital sulcus, calcarine sulcus and cingulate sulcus). As the brain continues to expand and grow, the cortex undergoes further folding to accommodate the increasing number of neurons and connections. This growth is partially influenced by mechanical forces exerted by the expanding brain tissue (Essen, D.C.v. 1997). Secondary and tertiary sulci form as a result of additional folding, creating a more intricate pattern of gyri and sulci (White et al. 2010; Armstrong et al. 1995; Zilles et al. 1989). From an anatomical point of view, deeper sulci yield SWMBs with higher curvatures, particularly those underlying primary and secondary sulci. In chimpanzees, especially in the lower temporo-occipital areas, the quasi lack of secondary and tertiary sulci explains the exclusive flat shapes identified, as illustrated in Fig. 16B, and provide evidence of the link between lower sulcal depth and associated lower SWMB curvature as compared with humans. The absence of secondary and tertiary sulci also reduces the amount of shorter fiber bundles that are present in humans, and could therefore be a possible hypothesis for the presence of globally longer fiber bundles in the chimpanzee brain compared to the human brain.

The relation between SWMBs and brain gyrification is particularly intriguing. This would deserve more exploration to understand the link between the sulcation profile and the underlying connections. If few previous studies have identified the anatomy of the gyral white matter (Dannhoff et al. 2023), inter-species differences in sulco-gyral patterns are prominent, posing a substantial challenge for robustly comparing their superficial structural connectivity networks, especially in brain regions known for high variability, such as the frontal lobe (Juch et al. 2005).

Interestingly, human-specific empirically classified 6- and J-shaped SWMBs were observed, primarily intersecting the insula. The presence of these patterns in SWMBs surrounding the insula has previously been noted in humans by Labra Avila (2020). Remarkably, studies investigating the insula from a neuro-evolutionary perspective are relatively scarce. One study has indicated that the insula structure underwent a significant expansion during human evolution (Kaas 2013). Additionally, in humans, the insula is known to be part of the brain regions exhibiting substantial growth during postnatal development (Hill et al. 2010), with the human insula being larger compared to that of apes and other primates (Semendeferi and Damasio 2000). Moreover, the insula seems to play a crucial role in complex processes such as taste perception, empathy, and social awareness (Keysers et al. 2010). The increased size of the human insula, its unique growth trajectory during human brain development, and its involvement in human-specific functions suggest the establishment of novel connections with different cortical regions required for processing these functions. A larger insular size could facilitate the extension of pathways crossing it to reach various brain regions. Last, the known restructuring of the human brain due to bipedalism, contrasting with the relatively unchanged position of the chimpanzee brain typical of a quadrupedal primate, might be the cause of twisting and rearrangement of the trajectory of white matter pathways connecting the insula.

Leveraging a point-cloud-based clustering algorithm, we identified key SWMB shapes, reducing the operator bias inherent in visual classification. These shapes primarily fall into two categories: flat and curved fiber bundles. The correspondence between the empirical and the isomap classifications was high for k = 2 in both species in defining these two morphologies. Flatter bundles seem predominantly located in the ventral region of the brain, while curved bundles are distributed more widely, especially in human subjects.

Among the curved fiber bundles, we distinguished four subtypes respectively corresponding to U-fiber bundles, C-fiber bundles, Open-U fiber bundles and V-fiber bundles. The empirical visual discrimination of the fiber bundles was supported by the results obtained with the automatic clustering method, at the exception of the V-fiber bundles that could not be deciphered as an individual cluster.

The lack of detection of V-fiber bundles by the isomap algorithm can be explained by two observations. First, these particular “V” fiber bundles highly resemble the Open-U fiber bundles. Indeed, both belong to the longest superficial bundles of the atlas, and depict a decussing of the fibers at their extremities giving them the aspect of a ‘fan’. Second, the V-shaped fiber clusters are highly underrepresented with respect to the others, making them hardly identifiable by the algorithm, merging them with the U-shaped fiber bundles. Interestingly, these particular shapes have already been noticed in other studies of the superficial connectivity in humans (Labra Avila 2020), and strikingly resembles the one found in our human SWMB atlas.

Various interpretations can explain the presence of U, Open-U, and curved shapes in the dorsal part of the chimpanzee brain. One of them is the progressive closure of fiber bundle edges from the ventral to the dorsal part of the brain. This accounts for flatter bundles in the inferior temporal, frontal, and occipital lobes, and more curved/closed edge bundles in the middle/superior frontal, parietal, and superior occipital lobes. The relative co-localization of the C, U and Open-U fiber bundles in the superior part of the different lobes also demonstrate the accuracy of our subgroup classification. Indeed, as seen for instance in the case of the frontal aslants, which is an associative bundle also considered superficial, the different subgroups of fibers composing it are supporting one another.

The classification of SWMBs can be approached from different perspectives, focusing on shape or loco-regional considerations, such as the number of sulci crossed. Here we primarily addressed white matter bundle shapes. In the case of the frontal aslant, our study aligns with the work from Catani et al. (2012) depicting different subdivisions of this bundle in the human brain, finding similar subdivisions in the chimpanzee brain. The U-fiber bundles are usually described in the human brain as fibers surrounding sulci (Meynert 1885), in our case, some fiber bundles were also observed spanning more than one sulci, which is particularly true concerning the inferior temporal lobe, and confirms the results from Shin et al. (2019). Interestingly, our study revealed almost symmetrical patterns of shapes and their brain localization between the two hemispheres, suggesting that superficial white matter connectivity, at least when considering SWMB shapes, may not exhibit significant asymmetries, in contrast to deep white fiber bundles, which can reflect lateralization of specific brain functions in some cases.

### Limitations

Despite significant advancements in diffusion MRI techniques for accurately mapping superficial connectivity, exploring SWMBs remains a great challenge. This challenge is particularly difficult to meet in the case of chimpanzees for which only few brain imaging databases are open to the neuroscientific community. Our study was only possible because we had access to such data. Nevertheless, the MRI data collected are not without limitations, primarily stemming from hardware constraints, including moderate resolution (on the order of 2 mm), and the presence of severe imaging artifacts such as eddy currents and susceptibility artifacts more pronounced in chimpanzees than in humans due to their specific anatomy.

These factors necessitated meticulous corrections but inevitably reduced the effective resolution of the data. In addition, local models of the diffusion process and fiber tracking methods also have their own limitations, missing some real fiber pathways or inducing false positives. Our SWMB atlas reconstruction pipeline was intentionally and specifically designed to minimize such errors. However, since no alternative resources currently exist for SWMBs (e.g., from Klinger’s dissections or polarized light imaging), we cannot definitively assert that the hundreds of SWMBs found in our chimpanzee and human atlases are entirely free from errors. Nonetheless, by applying the exact same pipeline to analyze chimpanzee and human connectivity, we aim to maintain a fair basis for comparison. Finally, it is worth noting that this study did not encompass an investigation of joint structural and functional connectivity due to the unavailability of bold-fMRI or paradigm-based fMRI datasets in the highly protected chimpanzee species.

### Perspectives

A potential avenue for enhancing our study’s robustness involves exploring the relationship between clusters of superficial white matter associative fascicles and the number of sulci they traverse. Evaluating the number of sulci crossed by a SWMB and relating this number to openness of the bundle shape could be a further way to subdivide SWMBs into specific classes of bundles crossing one, two or three sulci. This approach also offers an opportunity to develop new ontologies for SWMBs.

Currently, these ontologies are primarily limited to associating names of cortical areas connected by a SWMB, as provided in atlases like the DAVI130 for the chimpanzee brain (Vickery et al. 2020) or the Desikan and Destrieux atlases for the human brain (Desikan et al. 2006; Destrieux et al. 2010), with each bundle’s connectivity.

Considering the link between gyrification and SWMB, previous studies reported an evolution of the gyrification index with age in chimpanzees (Autrey et al. 2014). Thus, studying the age-related trajectory of short association fibers with related gyrification patterns would probably allow a better comprehension of the adult mature brain cortical and structural organisation.

Beyond the exploration of the short cortico-cortical associative fibers, it might be interesting to further extend this study to the exploration of the short connections populating the brainstem and cerebellum, which have been relatively underexplored in non-human primates.

### Conclusion

In this study, we conducted a comprehensive investigation into the morphological organization of SWMBs in chimpanzee and human brains, striving to compare their organizational and morphological characteristics. Notably, we emphasized the relationship between SWMB and brain gyrification, an aspect worthy of further exploration to understand the link between sulcation profiles and underlying connections. The presence of various subtypes within curved fiber bundles, including U, C, Open-U, and V shapes, reflects the complexity of SWMB morphology. Interestingly, certain SWMB shapes observed in this study resemble those found in human SWMB, suggesting potential evolutionary connections. While SWMB classification can be approached from various perspectives, focusing on shape contribute valuable insights into the morphological aspects of SWMB and their potential implications for human evolution and cognitive development.

## Figures and Tables

**Fig. 18:**
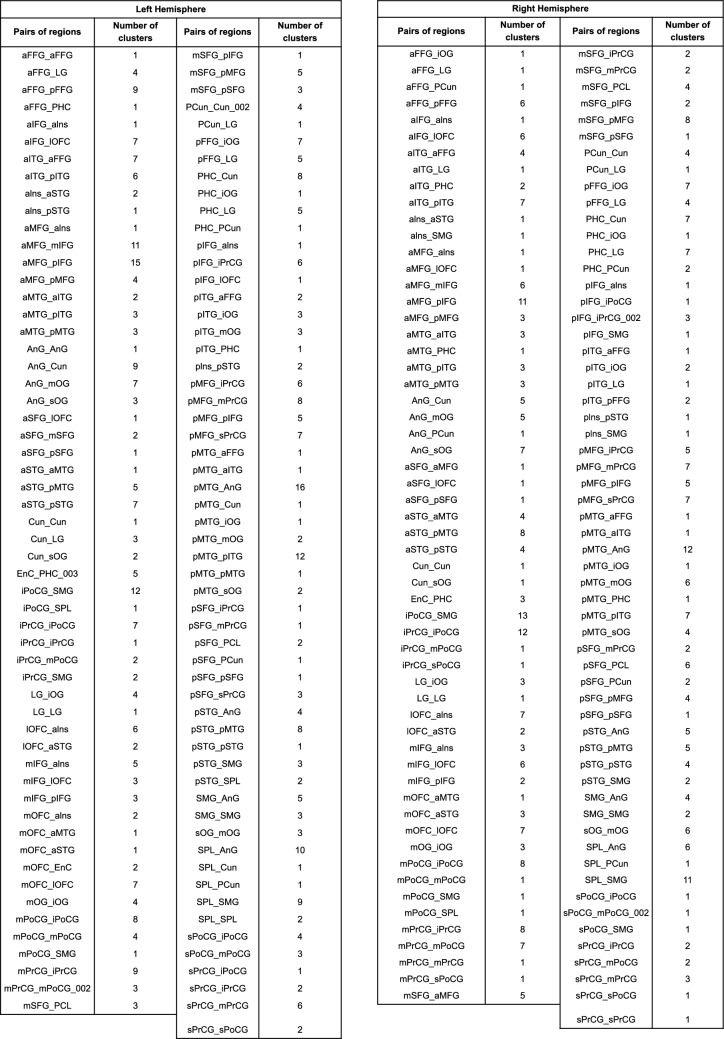
Table of corresponding cortical regions and number of clusters for the chimpanzee superficial white matter bundles

**Fig. 19:**
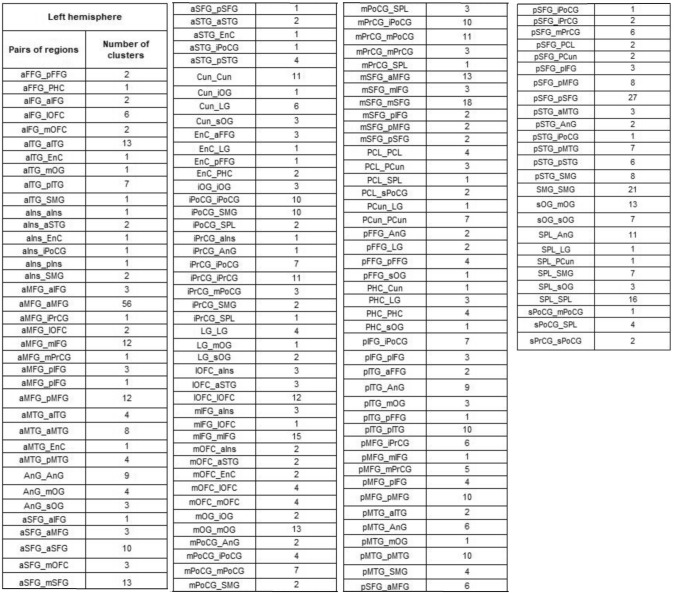
Table of corresponding cortical regions and number of clusters for the left human superficial white matter bundles

**Fig. 20:**
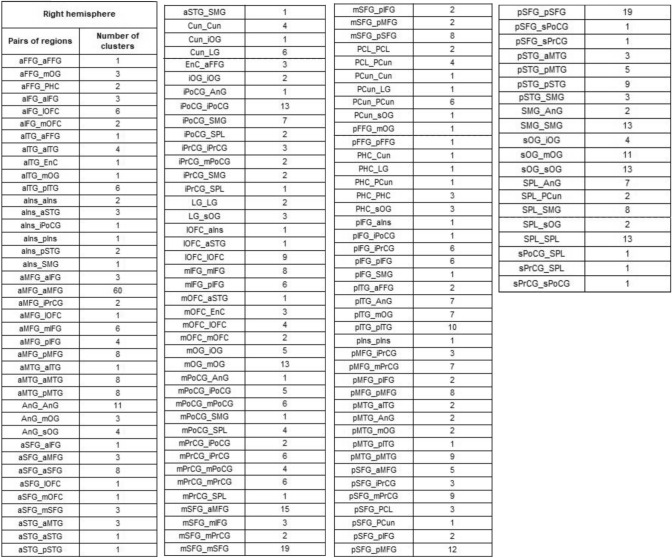
Table of corresponding cortical regions and number of clusters for the right human superficial white matter bundles

**Fig. 21:**
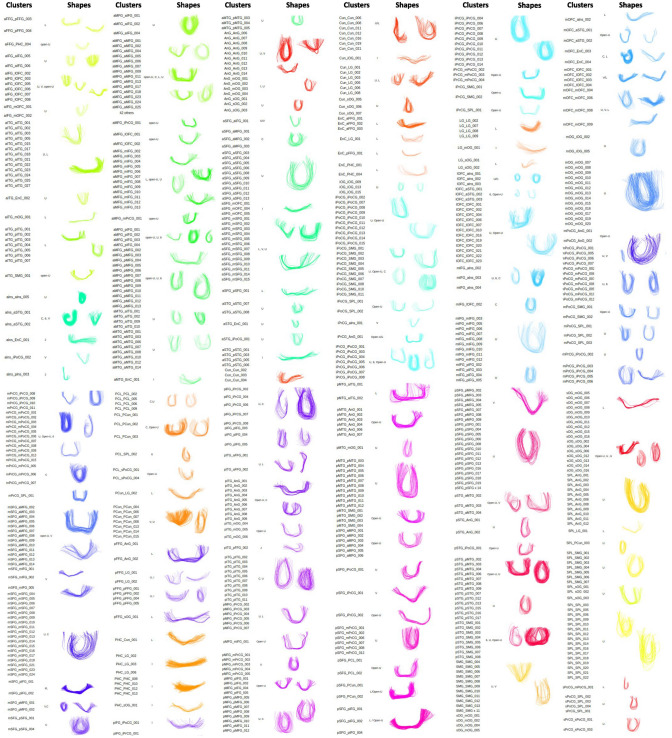
Bundles of the left SWMB atlas of the human brain and corresponding shapes

**Fig. 22:**
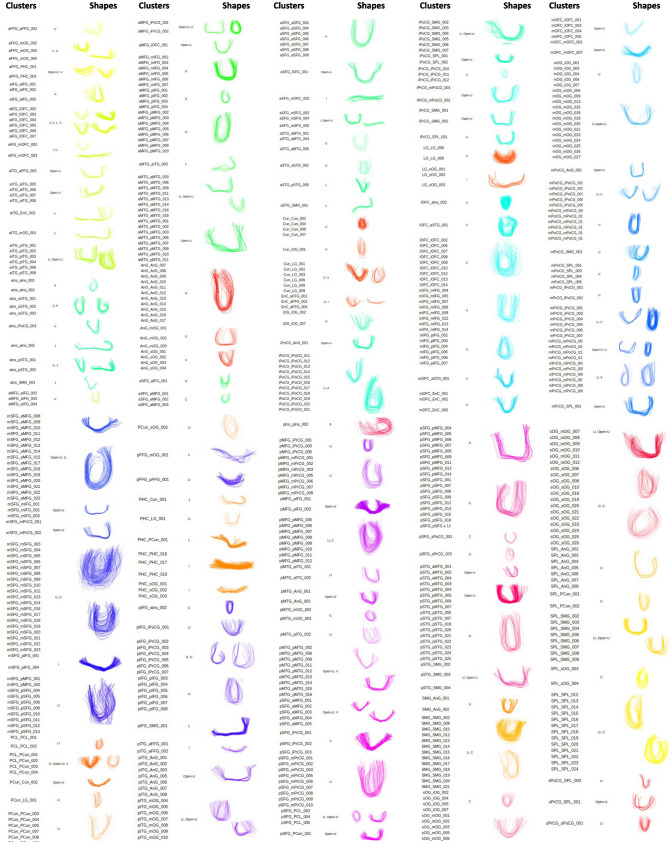
Bundles of the right SWMB atlas of the human brain and corresponding shapes

**Fig. 23:**
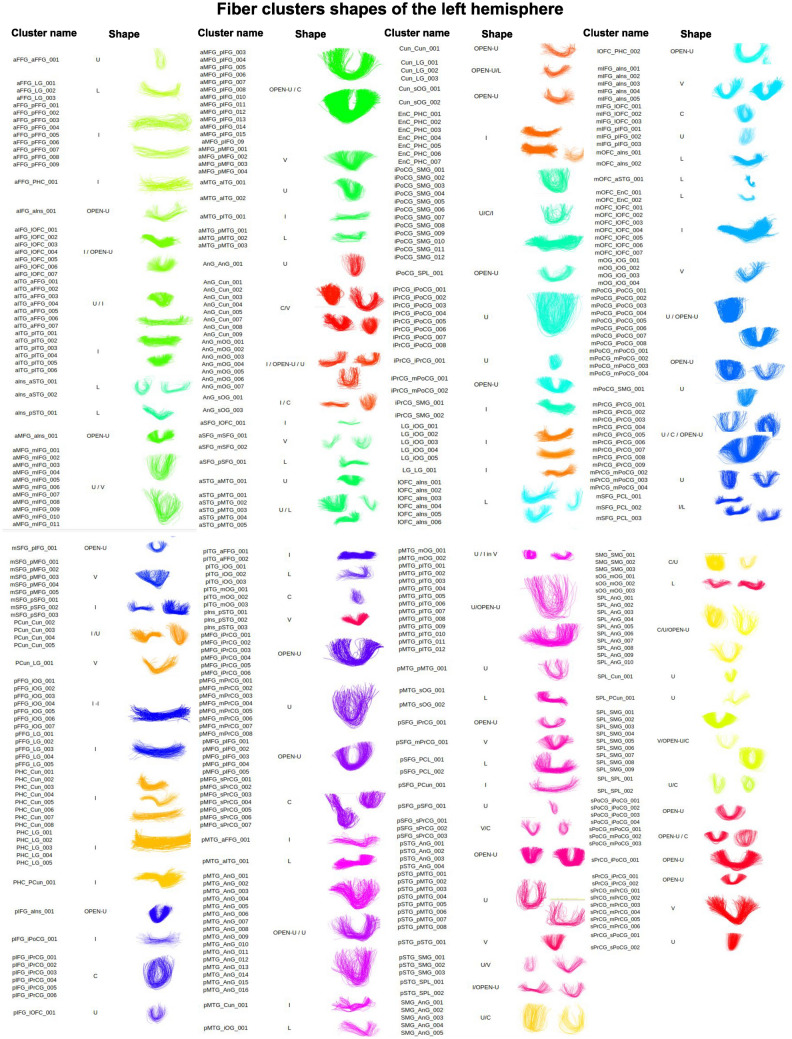
Bundles of the left SWMB atlas of the chimpanzee brain and corresponding shapes

**Fig. 24:**
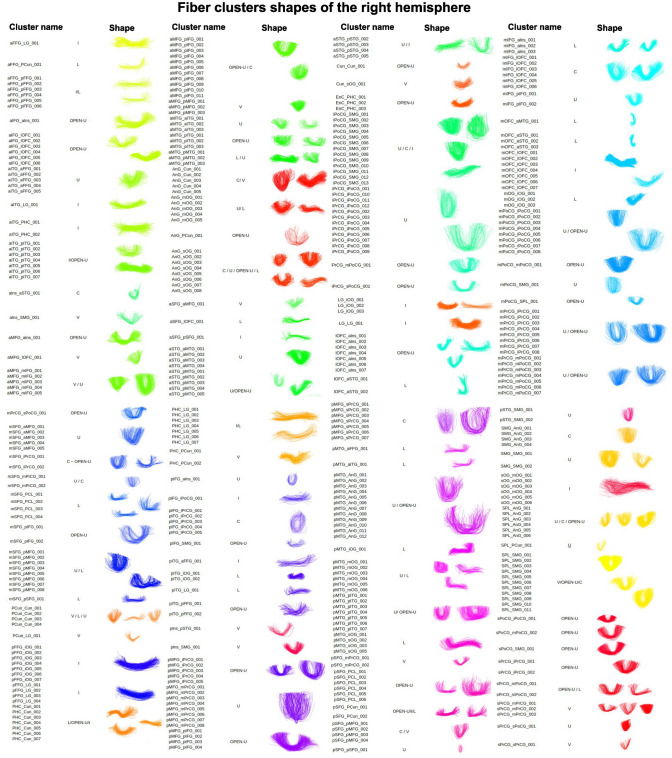
Bundles of the right SWMB atlas of the chimpanzee brain and corresponding shapes

**Fig. 25:**
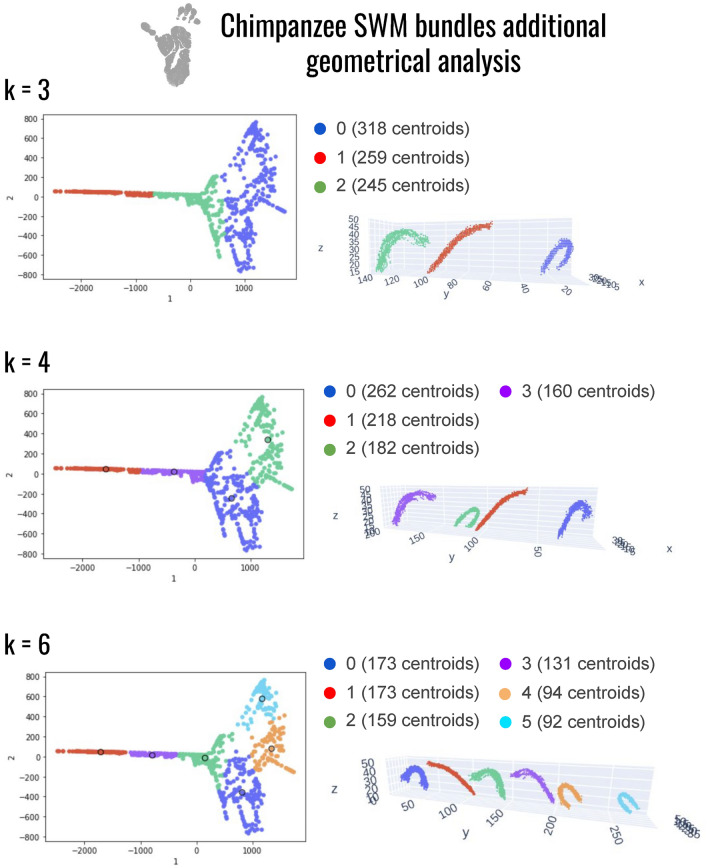
Additional morphological investigation of the superficial white matter bundles of the chimpanzee brain. Results are presented for k = 3 to k = 6, graphs are presenting points corresponding to each centroid point cloud with corresponding clusters. For each cluster, the number of centroids is indicated. Each color corresponds to a different cluster. Next to each graph is presented the average shape related to each cluster

**Fig. 26:**
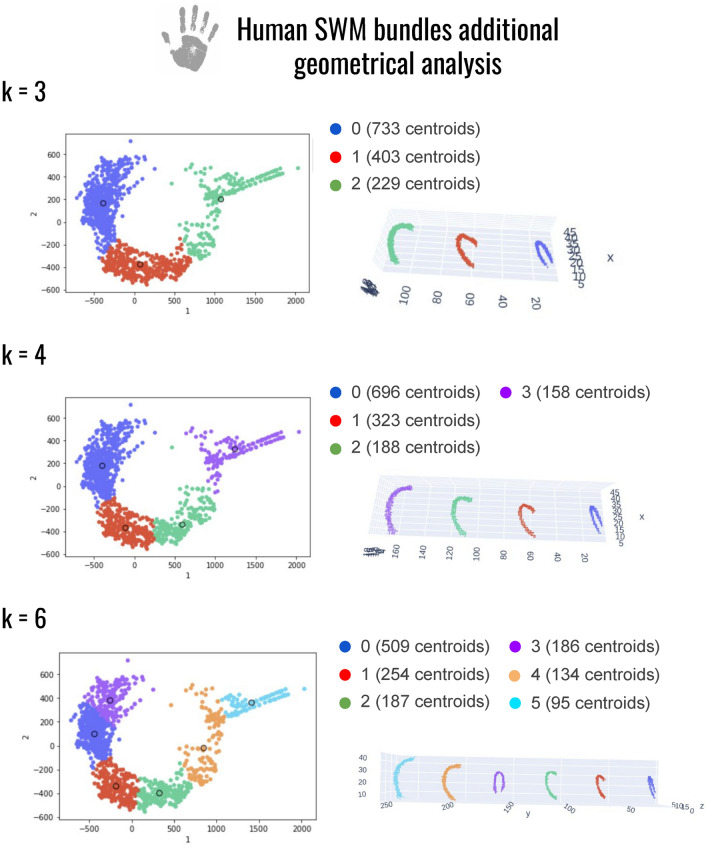
Additional morphological investigation of the superficial white matter bundles of the human brain. Results are presented for k = 3 to k = 6, graphs are presenting points corresponding to each centroid point cloud with corresponding clusters. For each cluster, the number of centroids is indicated. Each color corresponds to a different cluster. Next to each graph is presented the average shape related to each cluster

**Fig. 27:**
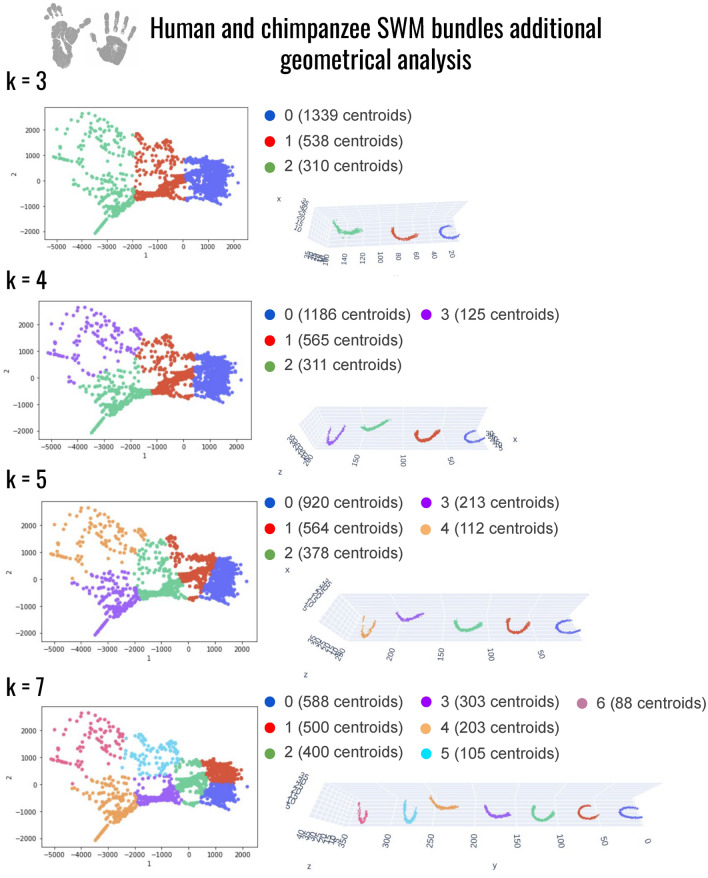
Additional morphological investigation of the superficial white matter bundles of the human and chimpanzee brains. Results are presented for k = 3 to k = 7, graphs are presenting points corresponding to each centroid point cloud with corresponding clusters. For each cluster, the number of centroids is indicated. Each color corresponds to a different cluster. Next to each graph is presented the average shape related to each cluster

## Data Availability

The chimpanzee SWMB atlas used in this study is available on the Zenodo platform at https://zenodo.org/record/7147503. The human SWMB is available on the Zenodo platform at https://zenodo.org/records/7308606. The construction of atlases is based on the analysis of the anatomical and diffusion MRI dataset using the tractography and fiber clustering tools available from the Ginkgo toolbox (Ginkgo Team, GAIA, BAOBAB, NeuroSpin, Paris-Saclay University, CNRS, CEA, https://framagit.org/cpoupon/gkg). The isomap algorithm used for this analysis can be found on the neurospin/github repository: https://github.com/neurospin/point-cloud-pattern-mining. The newly defined cortical human atlas, based on the Desikan-Killiany Atlas, matching that of the DAVI130 chimpanzee cortical atlas is available on the Zenodo platform at https://zenodo.org/uploads/11174211.

## Appendix

### A1 Corresponding number of clusters for each cortical region of the chimpanzee and human brains

See Figs. 18, 19, 20.

### A2 Corresponding SWMB and shape observed empirically

See Figs. 21, 22, 23 and 24.

### A3 Additional human and chimpanzee superficial white matter bundles geometrical analysis

The strategy used for the geometric analysis was to progressively increase the value of the number of clusters as long as the resulting clusters distinctly exhibited diverse shapes.

In chimpanzees, see Fig. 25, the value of k = 2 (as presented in the results section) delineated the distinction between curved and straight/flat shapes. Increasing it to k = 3 revealed a greater diversity of shapes, suggesting that higher k values may enhance shape differentiation. A similar trend was observed for k = 4. Notably, examining cluster 3 indicates the potential for extracting additional shapes with higher k values. Increasing k to 5, as outlined in the method section, provided relevant insights. However, elevating k to 6 resulted mainly in further subdivision of previously identified shapes, exemplified by clusters 4 and 5.

In humans, see Fig. 26, similar to chimpanzees, the value of k = 2 revealed two distinct shapes (flat versus enclosed edge bundles). We subsequently increased k to 3 and then to 4 in an attempt to differentiate flatter bundle shapes and variations in the curvature intensity of the fibers, which was achieved at k = 5. However, elevating k to 6 merely resulted in further subdivision of pre-existing bundle shapes, as evidenced by clusters 0 and 3.

When putting human and chimpanzee datasets all together, see Fig. 27, following the same logic as previously, continuously increasing the value for k led to the appearing of multiple bundle shapes until k = 7 where no further morphological/geometrical differentiation could be observed.
